# Supplementary material for: Kinetics of radiation-induced DNA double-strand breaks through coarse-grained simulations
Source: Biophys J. 2023 Jul 17;122(16):3314–22. doi: 10.1016/j.bpj.2023.07.008 (PMC10465705; doi:10.1016/j.bpj.2023.07.008)
Supplement: Document S2. Article plus supporting material [file mmc2.pdf]

Biophysical Journal
Article

Biophysical Society

Kinetics of radiation-induced DNA double-strand breaks through coarse-grained simulations

Manuel Micheloni,[1,2] Lorenzo Petrolli,[1,2] Gianluca Lattanzi,[1,2] and Raffaello Potestio[1,2,*]
[1]Department of Physics, University of Trento, Trento, Italy and [2]INFN-TIFPA, Trento Institute for Fundamental Physics and Applications, Trento, Italy

ABSTRACT Double-strand breaks (DSBs), i.e., the covalent cut of the DNA backbone over both strands, are a detrimental outcome of cell irradiation, bearing chromosomal aberrations and leading to cell apoptosis. In the early stages of the evolution of a DSB, the disruption of the residual interactions between the DNA moieties drives the fracture of the helical layout; in spite of its biological significance, the details of this process are still largely uncertain. Here, we address the mechanical rupture of DNA by DSBs via coarse-grained molecular dynamics simulations: the setup involves a 3855-bp DNA filament and diverse DSB motifs, i.e., within a range of distances between strand breaks (or DSB distance). By employing a coarse-grained model of DNA, we access the molecular details and characteristic timescales of the rupturing process. A sequence-nonspecific, linear correlation is observed between the DSB distance and the internal energy contribution to the disruption of the residual (Watson-Crick and stacking) contacts between DNA moieties, which is seemingly driven by an abrupt, cooperative process. Moreover, we infer an exponential dependence of the characteristic rupture times on the DSB distances, which we associate to an Arrhenius-like law of thermally-activated processes. This work lays the foundations of a detailed, mechanistic assessment of DSBs in silico as a benchmark to both numerical simulations and data from single-molecule experiments.

SIGNIFICANCE Ionizing radiation deals a plethora of lesions on a DNA filament, the rupture of the covalent bonds over both strands (or double-strand breaks [DSBs]) being the most detrimental for the cell survival. Yet, in the earliest stages of the evolution of a DSB, noncovalent interactions hold the DNA moieties, thereby inhibiting/delaying the rupture of the DNA helix. Here, we assess the fracture kinetics of DNA by different DSB motifs via extensive coarse-grained molecular dynamics simulations. The characteristic rupture times increase exponentially with the distance between strand breaks, following an Arrhenius-like law of thermally-activated processes. This work contributes a detailed and quantitative picture of the earliest stages of radiation-enforced DNA lesions and lays the foundations of a bottom-up characterization of the cell survival at a broader scale.

INTRODUCTION

DNA holds the hereditary information required by cells to replicate and carry out their biological activity. Cells, however, are constantly threatened by a variety of toxic agents, from both endogenous processes, i.e., naturally associated with a cell life cycle (e.g., as by-products of metabolic stresses (1) and DNA replication (2)) and exogenous vectors, such as chemicals and ionizing radiations (IRs) (3,4). In fact, IR accounts for a plethora of chemical and mechanical lesions of the DNA molecule, involving a wide range of space- and timescales, which are mediated by a cascade of inelastic collisions and radiolytic reactions (5). Among the diverse radiation-enforced DNA lesions, however, the disruption of the phosphoester backbone over both DNA strands, or a double-strand break (DSB), is arguably the most toxic (3). DSBs account for major cytotoxic effects, and critically so at sites of highly-condensed lesions within localized DNA volumes (6,7). Indeed, lesion clustering is a striking lethal feature of IR at high linear energy transfer regimes, i.e., associated with a massive release of energy at the microscale (8,9). From a radiation biology perspective, a key observable via the irradiation of cell-lines in vitro is defined by the cell survival ratio S upon the radiation dose D—the latter being the energy deposited per unit of mass. A simplified, yet effective, theoretical framework that properly describes S as a function of D is the so-called linear-quadratic model whereby $S = e^{-\alpha D} e^{-\beta D^2}$: here, α and β are associated

Submitted July 11, 2022, and accepted for publication July 12, 2023.
*Correspondence: raffaello.potestio@unitn.it
Editor: Margaret Shun Cheung.
https://doi.org/10.1016/j.bpj.2023.07.008
© 2023 Biophysical Society.
This is an open access article under the CC BY license (http://creativecommons.org/licenses/by/4.0/).

with the cell radiosensitivity and depend on a variety of factors, namely the cell type, the stage of the cell cycle, the dose fractionation regime, the radiation quality and energy, and so on (10). While α and β are empirically defined, several phenomenological (11) and microdosimetric models (12,13) have attempted at establishing a mechanistic foundation of the cell survival, relying upon a microscopic description of the layout of the events of energy deposition about a radiation carrier. In this respect, Monte-Carlo track structure (MCTS) codes have been widely employed to characterize in silico the evolution of a radiation field as a stochastic sequence of (elastic, inelastic) collisions with a medium—such as a water continuum, in a cell-like scenario—at a resolution level that is hardly accessible by conventional experimental techniques (14–16). Effective criteria have thus been established to associate the local distribution of physical and chemical processes (or the radiation track) with the early occurrence of "lethal" DNA lesions, such as DSBs, within significant subcellular volumes (17–19). Overall, these techniques depict the irradiation of cells via a statistically robust, mean-field approach, as multiple replicas of the track are averaged. However, the theoretical framework underneath MCTS codes overlooks the dynamical detail to characterize the subsequent evolution of DSBs at the molecular level. In fact, according to a widely accepted paradigm, DSB motifs are implicitly deemed to be effective, achieving a nonreversible rupture of the DNA backbone, within an arbitrary distance of the two strand nicks (17,18,20). Yet, it was earlier inferred that the distribution of DSB motifs is likely associated with a radiation quality, i.e., DSBs occur with a different likelihood upon the distance between cuts (21). Moreover, the disruption of the residual contact interface holding the DNA moieties should arguably contribute a unique (thermodynamic, kinetic) bias on the local thermal and mechanical stability of DNA varying upon the DSB motif. A complementary insight might thus be offered by molecular dynamics (MD) techniques, evolving the transient dynamics of DSBs over single DNA molecules. Recently, Landuzzi and coworkers (22) have assessed the thermal and forceful fracture of DNA by DSBs on a 31-bp DNA sequence depicting a short DNA linker (i.e., the exposed, linear DNA stretch between nucleosomes in a chromatin fiber) by all-atom MD. Notably, they inferred that the direct observation of the rupture of DNA by DSB motifs beyond a distance of 3 bp is unlikely within the timescales associated with the activity of the DNA damage repair machinery. However, atomistic MD is bound by a significant numerical overhead, so the accessible dynamics of biological systems is restricted to a few microseconds and/or a few replicas—which might bias its statistical significance in quantitative assessments. These limitations may be circumvented by exploiting coarse-grained (CG) models (23–25), whereby groups of atoms are specifically mapped into a lower-resolution description of the system: the CG sites interact by means of effective potentials, defined by either *top-down* or *bottom-up* approaches (26,27). In this work, we employ the oxDNA CG (25) force field to assess the rupturing process of DNA driven by DSB lesions. We enforce the twofold nick of the DNA backbone involving no local base modifications or deletions (or T1-DSBs, as described by Schipler and Iliakis (3)) on a 3855-bp DNA molecule, kept at varied end-to-end distances by fixed forces. Here, DSBs are described within a simplified, effective framework, by removing the covalent interactions between adjacent CG nucleotides, varying the distance between strand breaks (0–3 bp). The setup resembles an optical tweezers scenario, with the DNA being adsorbed and/or fixed at one end of the molecule and slightly stretched by a constant force regime at the other end, taking advantage of an established (experimental, theoretical) framework in single-molecule experiments on nucleic acids (28,29).

We thus perform extensive MD simulations, tracking the transient dynamics of the fractured DNA moieties and the kinetics of the subsequent rupturing process, which is driven by the disruption of all residual contacts holding the helical framework. The trend we observe tallies with a depiction of the DNA rupturing by DSBs as a cooperative, thermally-activated process, characterized by an exponential increase of the average rupture times with the distance between strand breaks. Finally, we discuss our observations in terms of an Arrhenius law and propose a protocol to rescale the times of the CG dynamics by the (experimental, in silico) diffusion coefficients of freely-diffusing DNA.

MATERIALS AND METHODS

System model and force field

We simulated a 3855-bp, double-stranded DNA sequence in the B conformation (see supporting material section S1), adapted from the optical tweezing experimental setup of Wang and co-workers (30). We employed the oxDNA2 CG force field, which has been acknowledged to faithfully reproduce both the thermodynamic and the mechanical features of the DNA double helix (31). The earliest releases of the model (25,32) take into account temperature-dependent, sequence-specific stacking interactions and Watson-Crick hydrogen bonding between nucleotides. oxDNA2 improves on the original model, effectively involving features of the major and minor grooves of B-DNA and the salt-dependent modulations of the force field via Debye-Hückel interactions, thereby refining the behavior of DNA in implicit solvent and diverse concentrations of monovalent salt, down to a physiological environment (31). Moreover, the level of resolution associated with oxDNA2 is adequate to detail the dynamical features of DSB lesions while lowering the numerical overhead of the atomistic force fields, hence covering time frames that are biologically significant (26,33).

MD simulations

MD simulations were carried out operating on the LAMMPS software platform (34). The dynamics of the DNA molecules was assessed in the NVT ensemble at $T = 310$ K and a monovalent salt concentration of 0.15 M, matching a physiological environment. All simulations were performed employing the rigid-body Langevin-type integrator (Langevin C) (35), which solves the equations of motion accounting for an implicit solvent background. Indeed, this integrator has shown enhanced performance,

in contrast to the standard LAMMPS Langevin algorithm for DNA simulations, while allowing a stable integration of the equations of motion at larger time steps (33,36)—which we set to $\Delta t = 5 \times 10^{-3}\ \tau$, together with a damping coefficient of the Langevin thermostat $\zeta = 2.5\ \tau$. Thus, we carried out a step-wise thermalization protocol (see supporting material section S2) in order to obtain equilibrated DNA conformations at varied target elongations—namely, $R_{ee} = 1000$, 1100, and 1200 nm, with R_{ee} as the DNA end-to-end distance. These conformations are 76%, 84%, and 92% of the contour length L ($\approx$1310 nm) of the chain. Indeed, the desired (average) elongation ratios were maintained by applying a harmonic potential to one end of the DNA molecule, with harmonic constant $k = 5.7$ N/m, and an external force $F_z = 0.42, 0.88, 3.06$ pN to the terminal nucleotides at the opposite end, derived as follows (37):

$$\frac{F\xi}{k_B T} = \frac{1}{4}\left(1 - \frac{R_{ee}}{L}\right)^{-2} + \frac{R_{ee}}{L} - \frac{1}{4}, \tag{1}$$

with $\xi \approx$ 50 nm the persistence length of DNA. Such conformations are compliant with a low-force regime ($\leq$ 5 pN), i.e. where DNA reacts by entropic compliance to the applied tension and the Marko-Siggia formulation of the worm-like chain model is applicable (30,38). The three configurations of the DNA helix served as starting frames of a set of classic MD replicas aimed at assessing the early effects of DSBs. As the radiation-enforced lesions of DNA bear several levels of complexity varying upon the resolution scale (3,16), we adopt a simplified, effective framework to characterize the local strain from the disruption of the DNA backbone that both faithfully depicts the process and is numerically feasible. Indeed, we focused on T1-DSBs as described by Schipler and Iliakis (3), where the phosphoester bonds of the DNA backbone are disrupted. We thus neglect all modifications involving the nitrogenous bases, and allow thermal fluctuations to detach the DNA moieties. We hereby define the DSB distance b_d as the distance in between the twofold nicks of the DNA backbone, quantified in number of native base pairs (Fig. 1). Diverse DSB motifs were enforced by removing the covalent bonds between adjacent nucleotides on both DNA strands at distances $b_d = 0$, 1, 2, and 3, about half way of the DNA filament (see supporting material section S1): in LAMMPS, this is implemented by deleting the bond ID between the nearest neighboring nucleotides in the topology file. For each value of b_d, numerous independent replicas were run from the same starting configurations. We remark that no prior minimization/thermalization protocol of the DSB scenarios was carried out, to depict the subsequent dynamics of a DNA molecule broken abruptly by the radiation field. A snapshot of a DNA rupture by a DSB at $b_d = 1$ is shown in Fig. 1.

Derivation of the characteristic times of the DNA rupture by DSBs

The characteristic time associated with the rupture of the DNA moieties τ_{b_d} was estimated for all replicas of each (b_d, F_z) DSB scenario via a twofold procedure. We first established a geometrical, heuristic criterion to unequivocally frame the DNA-fracturing event based upon the minimum distance between the broken DNA moieties (see supporting material section S3 for details): the specific choices were corroborated by visual inspection of the MD trajectories, so that we could focus on a time frame about the rupturing event. Likewise, we derived τ_{b_d} from the internal energy profile of the nucleotides involved with the residual contact interface between DNA moieties at the site of a DSB lesion, that is 1) the nucleotides lying in-between the twofold break of the DNA backbone and 2) the nucleotides closely overlooking the DSB interface on both sides of the lesion (see Fig. 1). Fig. 2 shows the internal energy contribution of the contact nucleotides at a blunt DSB (i.e., at $b_d = 0$) within a time span involving the rupture of the DNA molecule.

By inferring that the rupture of DNA by DSBs is arguably a two-state process (as observed by the internal energy profiles) and that the barrier

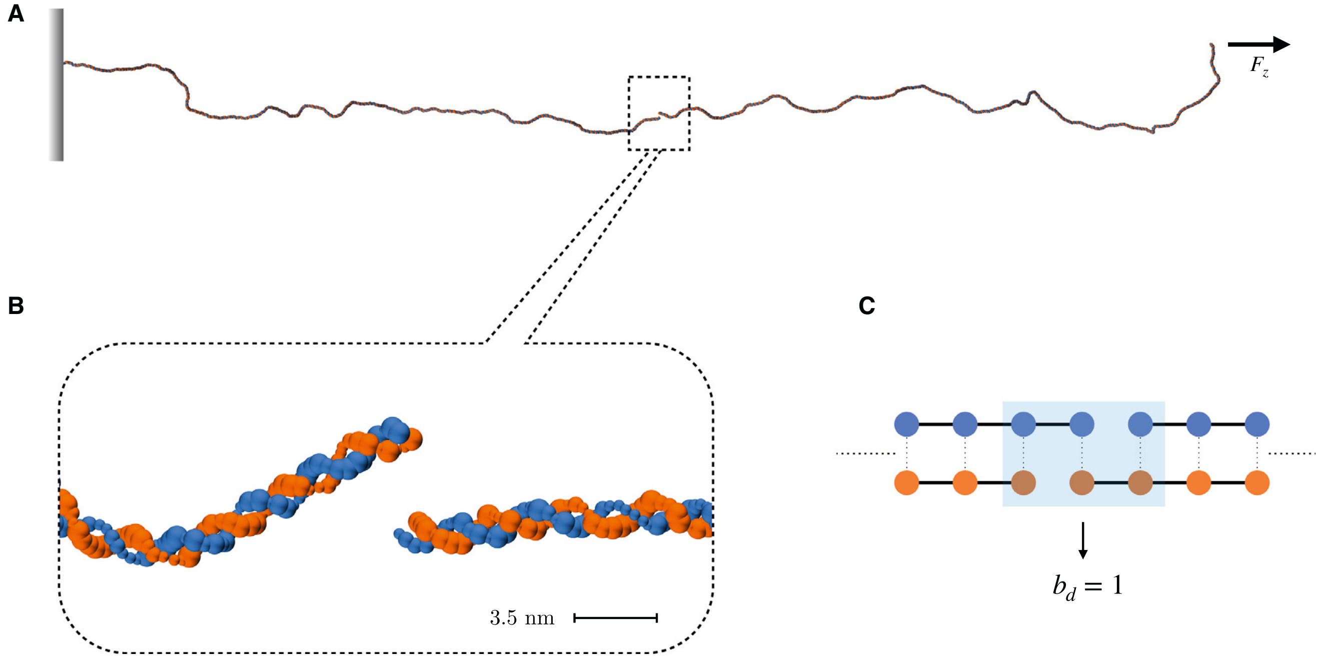

FIGURE 1 (*A*) Snapshot of the 3855-bp B-DNA molecule associated with an end-to-end distance of $\langle R_{ee} \rangle = 1000$ nm, broken by a double-strand break at distance $b_d = 1$—shown in detail in the inset (*B*). F_z is the constant external force acting on the DNA, computed according to Eq. 1—details are in the text. (*C*) Schematic depiction of a DNA double-strand break: the backbone connectivity is shown as solid lines, and hydrogen bonds between DNA strands are shown as dotted lines. The blue shaded region highlights the nucleotides involved with the residual contact interface between the DNA moieties. To enforce a double-strand break, covalent bonds are removed between adjacent nucleotides from both DNA strands. To see this figure in color, go online.

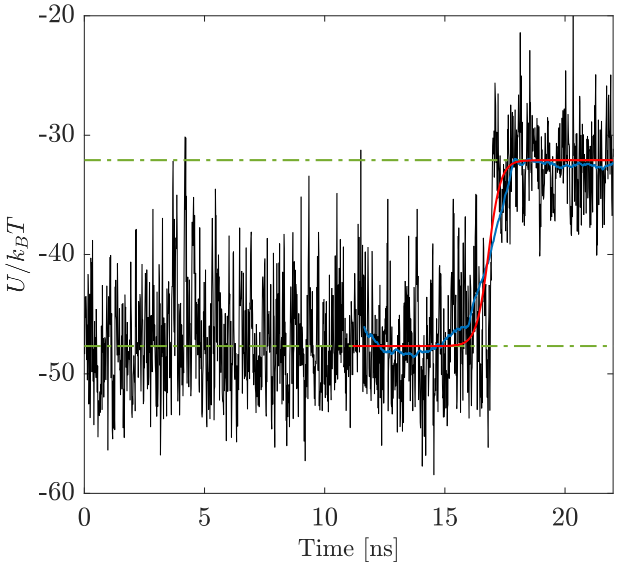

FIGURE 2 Profile of the local internal energy contribution from the nucleotides involved with the residual contact interface between broken DNA moieties, associated with the DNA rupture by a blunt DSB ($b_d = 0$) and an average end-to-end distance of the DNA filament $\langle R_{ee} \rangle = 1000$ nm. The lesion destabilizes the helical layout, driving a conformational transition of the DNA molecule from a bound to a broken state (lower and upper energy plateaus, shown as *dashed green lines*). The moving average of the potential energy profile (*blue line*) is fitted by a sigmoid curve (*red line*), whereby we assess the characteristic time and an internal energy contribution to the DNA rupture. To see this figure in color, go online.

dividing the bound (metastable) and broken thermodynamic basins is symmetrical at the peak, we performed a sigmoidal fit on the internal energy profile as

$$E_{\text{fit}} = E_{\min} + \frac{E_{\max} - E_{\min}}{1 + 10^{m(t_{50} - t)}}, \tag{2}$$

and derived the characteristic time of the DNA rupture at the flex of the curve. Here, $E_{\min}$ and $E_{\max}$ are associated with the lower and upper plateaus of the internal energy (characterizing the bound and broken states of the DNA molecule, respectively), and m is the steepness of the curve. The values of $\tau_{b_d} = t_{50}$ are estimated at the flex of the internal energy transition and closely match those derived by the heuristic criterion, thus validating ΔU as an effective proxy to frame the kinetics of the process. As a consequence, we implicitly estimate the internal energy contribution to the process as the difference between the internal energy at the flex of the sigmoid and at the lower-energy plateau—which we will tentatively associate with the activation state of the DNA rupture (vide infra). Additional and technical details about the fitting protocol are reported in supporting material section S3.

RESULTS AND DISCUSSION

As the structure of the DNA double helix is compromised by a DSB lesion, non-covalent interactions hold the broken DNA moieties: the system is thus confined to a metastable, bound state until the kinetic barrier associated with the rupture of the residual contact interface between the DNA moieties is overcome by means of stochastic thermal fluctuations.

Kinetics of the DNA fracture by DSB motifs

We firstly assessed the kinetics of the DNA fracture from the diverse DSB motifs by estimating the average rupture times $\overline{\tau}_{b_d}$ over all independent MD replicas associated with a given (b_d, F_z) combination—the results are outlined in Table 1.

The estimates of the average rupture times $\overline{\tau}_{b_d}$ seemingly fit an exponential law of the DSB distance—or, equivalently, a linear function of b_d as

$$\ln \overline{\tau}_{b_d} = A + k_1 b_d, \tag{3}$$

with A and k_1 coefficients from the fitting procedure—reported in Table 2 and shown in Fig. 3.

This trend replicates the exponential increase with size observed by Sengar and co-workers for the dissociation kinetics of short DNA duplexes, employing an earlier version of the oxDNA force field, thereby characterizing the rupture of DNA by DSBs as a rare event (33).

Notably, our data are similarly well fit by an alternative law of the form $\overline{\tau}_{b_d} = 10^{w_1 + w_2 b_d}$ (see supporting material section S6), which has been reported for the apparent thermal off-rates from the forced melting of DNA duplexes by atomic force spectroscopy, extrapolated in the limit of zero force (39–41). In fact, by assuming the rupture of DNA by DSBs being a cooperative, thermally-activated process (as similarly described in (40,41) for the denaturation of DNA), it is tempting to redefine the (A, k_1) coefficients of the exponential law (Eq. 3) in terms of Arrhenius-like factors as

$$\ln \overline{\tau}_{b_d} = \ln \overline{\tau}_0 + (\beta \Delta\Delta G^{\ddagger}) b_d, \tag{4}$$

with $\overline{\tau}_0$ as the average fracture time of DNA by blunt DSBs (i.e., at distance $b_d = 0$) and, in a broader sense, the kinetic contribution from the rupture of (stacking, electrostatic) interactions to the activation barrier; $\beta^{-1} = k_B T = 0.62$ kcal/mol at 310 K; and $\Delta\Delta G^{\ddagger}$ is a sequence-nonspecific, additive contribution from the disruption of Watson-Crick hydrogen bonds to $\Delta G^{\ddagger}$ (i.e., the activation free energy of the rupturing of DNA by the diverse DSB motifs), the latter thus increasing linearly with b_d. As shown in Table 2, $\Delta\Delta G^{\ddagger}$ ranges between 1.6 and 1.9 $k_B T$ among all scenarios, which is slightly higher than the values reported in references (39,41,42) for the activation free energy associated with the denaturation of DNA: arguably, this is accounted for by a lower entropic contribution from the local fraying of the DNA termini, the latter being dampened by the residual π-stack interactions between the DNA moieties in the current scenario. As expected, $\overline{\tau}_0$ is in line with the estimates of the average rupture times from blunt DSB motifs (see Table 1).

It might be argued that the application of a constant force regime enhances the kinetic rates of the DNA rupture by lowering the activation free energy barrier along the reaction coordinate of the process (39,41,43). Yet, as shown in Fig. 3 (and further discussed in supporting material section S7), the average rupture times $\overline{\tau}_{b_d}$ exhibit a negligible

TABLE 1 Structural, energetic, and kinetic properties of the various DSB distances for the different DNA setups under examination

F_z [pN]	$\langle R_{ee} \rangle$ [nm]	$\Delta\overline{U}/k_BT$				$\overline{\tau}$ [ns]			
		$b_d = 0$	$b_d = 1$	$b_d = 2$	$b_d = 3$	$b_d = 0$	$b_d = 1$	$b_d = 2$	$b_d = 3$
0.42	1000	8 ± 2	14 ± 4	23 ± 4	27 ± 6	1 ± 1	6 ± 6	105 ± 102	139 ± 123
0.88	1100	7 ± 2	14 ± 4	24 ± 4	28 ± 6	0.8 ± 0.7	4 ± 4	105 ± 104	138 ± 120
3.06	1200	8 ± 2	14 ± 4	24 ± 4	27 ± 4	0.9 ± 0.9	6 ± 7	88 ± 89	114 ± 102

Left: internal energy contribution to the DNA rupture as function of the DSB distance b_d and applied force, the latter being associated with an average end-to-end distance of the DNA molecule $\langle R_{ee} \rangle$. *Right*: average rupture times $\overline{\tau}_{b_d}$ from the different DSB motifs and DNA extensions: each value of $\overline{\tau}_{b_d}$ is estimated by averaging over all independent MD replicas. The characteristic times are scaled according to a straightforward conversion of LJ into SI units (33). In all cases, standard deviations are on the order of the average values due to the statistical nature of the process (see supporting material section S5).

dependence upon the tensile protocol enforced on the DNA molecule. In fact, the range of forces applied (and fluctuations thereof) lies within the threshold for a purely entropic response of the DNA molecule under tension (30,37,38) and below the mean contribution from the thermal bath (see supporting material section S6.1). Moreover, the forces exerted at the DNA termini quickly decorrelate along the chain (see supporting material section S6.2), whereby we infer that thermal fluctuations eventually account for the rupture of the DNA moieties.

Internal energy contribution to the rupture of DNA

We then focused on the internal energy contribution to the rupture of DNA by the DSB motifs and on its dependence on the DSB distance b_d. We adopted a consistent criterion (see supporting material section S3) to define $\Delta\overline{U}(b_d)$ as the average value of the difference between the internal energy of the system at τ_{b_d}—as described earlier in this work—and the internal energy plateau of the bound basin (values are reported in Table 1). Subsequently, we performed a linear fitting as

$$\frac{\Delta\overline{U}(b_d)}{k_BT} = \alpha b_d + \varsigma, \tag{5}$$

with α and ς as empirical coefficients—reported in Table 3.

In fact, the validity of this assumption, i.e., the existence of a linear correlation between the internal energy contribution to the rupture of DNA and the DSB distance b_d, is verified for all DNA conformations, as shown in Fig. 4. So far, our assessment has thus strongly hinted that the rupture of DNA by DSBs might be depicted effectively by a cooperative, abrupt transition between a metastable, bound state (held by residual hydrogen bonds and stacking contacts) and a broken state of the DNA moieties drifting from the lesion site. Moreover, this is arguably an activated process,

TABLE 2 A ($\overline{\tau}_0$) and k_1 ($\beta\Delta\Delta G^{\ddagger}$) coefficients from the fitting of the average rupture times $\overline{\tau}_{b_d}$ on the DSB distances b_d (Eqs. 3 and 4) for each (b_d, F_z) DNA scenario

F_z [pN]	A ($\overline{\tau}_0$) [ns]	k_1 ($\beta\Delta\Delta G^{\ddagger}$)
0.42	1.0 ± 1.0	1.7 ± 0.4
0.88	0.9 ± 0.7	1.8 ± 0.4
3.06	1.0 ± 1.0	1.7 ± 0.4

well described by transition state theory (44–47), according to which a system progresses between conformational basins across a dynamical bottleneck enforced by the transition state (‡). Given $\Delta G^{\ddagger}$ as the height of the activation free energy barrier associated with the rupturing process (see Fig. 5), we thus have

$$\Delta G^{\ddagger} = \Delta U^{\ddagger} - T\Delta S^{\ddagger}, \tag{6}$$

with $\Delta U^{\ddagger}$ and $\Delta S^{\ddagger}$ defining the internal energy and entropic contributions, respectively.

Taking into account the restraints enforced on the conformational freedom of the DNA molecules, we expect the entropic contribution to be negligible up to the peak of the free energy barrier, thus reducing Eq. 6 to

$$\Delta G^{\ddagger} = \Delta U^{\ddagger}. \tag{7}$$

As the barrier is overcome, the DNA moieties acquire a significant conformational freedom, thereby abruptly increasing the entropy of the system—Fig. 5 schematically illustrates this process. In light of these assumptions, we tentatively simplify and redefine $\Delta U^{\ddagger}$ by the internal energy contribution to the DNA rupturing process. Yet, $\Delta\overline{U}(b_d)$ hardly applies as an effective proxy of the activation free energy: in fact, the ratio between α and $\Delta\Delta G^{\ddagger}$ (both representing an additive contribution to the activation energy—see Eqs. 5 and 4, respectively) is about fivefold. Arguably, this is accounted for by a misestimate of the activation barrier, likely lying closer to the plateau of the metastable, bound state than to the flex of the sigmoidal fit of $\Delta U(b_d)$. Indeed, the rupture of DNA is driven by an abrupt transition so that small uncertainties in the estimate of τ_{b_d} reflect wide variations in the internal energy contribution (see Fig. 2). Moreover, the assumption of negligible entropy, which is valid up to the peak of the free energy barrier, requires an independent validation through refined MD techniques aimed at characterizing the reaction coordinate and the transition state—which is the objective of ongoing work.

Correlation between physical and simulation time in CG models

It is well acknowledged that CG models enhance the dynamics of systems with respect to their all-atom counterparts (26,48,49). This is inherent to the integration of

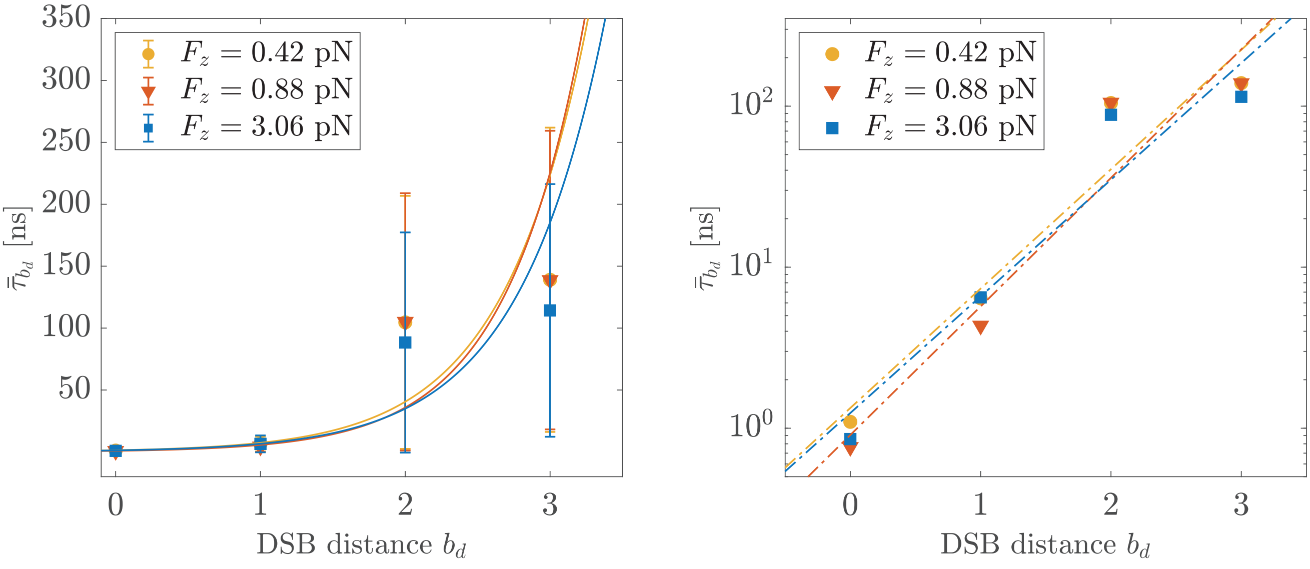

FIGURE 3 Average rupture times $\bar{\tau}_{b_d}$ as function of the DSB distance b_d (shown in semi-logarithmic scale in the *right panel*). The solid and dashed lines are defined upon the fitting protocol described by Eq. 3. To see this figure in color, go online.

several degrees of freedom, whereby the coarse-graining procedure somewhat smoothens the free energy landscape, thus favoring the transition between local free energy minima. Furthermore, CG models (such as oxDNA) are frequently implemented with implicit solvents, which lack a proper characterization of hydrodynamic effects from the collective motion of the solute particles nearby (50,51). These limitations only marginally, if at all, affect the characterization of the structural features of a system at equilibrium (30,52); they become critical, however, to the dynamical assessment of kinetic observables. Under the assumption that the modifications induced by the coarse-graining protocol are quantifiable in terms of a global redefinition of the characteristic times of a system (48), these issues might be tackled by rescaling the time steps of the CG simulation to match the outcome from a reference scenario, such as all-atom MD or wet-lab experiments. In this view, we related the timescales of the physical (experimental) process and those of its in silico (CG) depiction via the ratio of the diffusion coefficients of double-stranded DNA molecules $\Gamma(\zeta)$ as

$$t = \Gamma(\zeta)t_{\mathrm{CG}} = \frac{D_{\mathrm{CG}}(\zeta)}{D_{\mathrm{EXP}}}t_{\mathrm{CG}}, \tag{8}$$

where t is the best estimate of the time of the physical process, t_{CG} is the time of the CG simulation, and $D_{\mathrm{CG}}(\zeta)$ and D_{EXP} are the CG and experimental diffusion coefficients of DNA, respectively. In fact, a rescaling protocol based on the diffusion coefficients of DNA was adopted by Ouldridge and co-workers alike, who explored the hybridization kinetics of DNA oligomers (53). To estimate the value of $D_{\mathrm{CG}}(\zeta)$ for the freely-diffusing 3855-bp DNA molecule, we performed a MD simulation of $5 \times 10^6\ \tau$ in the NVT ensemble at $T = 310$ K, starting from the equilibrated, 1000-nm conformation of DNA. The value of

TABLE 3 α and ς coefficients from the linear fitting of the average internal energy contribution to the rupture of DNA $\Delta\bar{U}(b_d)$ upon the DSB distance, as described in Eq. 5, at different values of the external force

F_z (pN)	α	ς
0.42	7.1 ± 1.5	7.8 ± 1.6
0.88	7.5 ± 1.6	7.0 ± 1.9
3.06	6.7 ± 1.4	7.9 ± 1.7

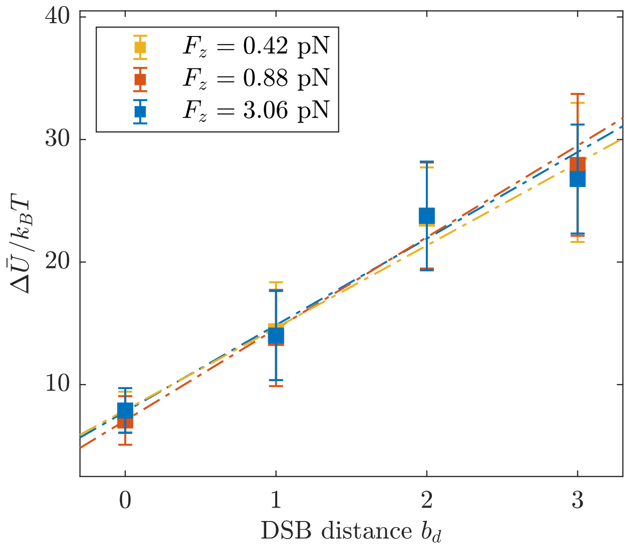

FIGURE 4 Average internal energy contribution to the rupture of DNA as function of the DSB distance b_d for each force regime. The dashed curves are obtained from the fitting protocol described by Eq. 5. To see this figure in color, go online.

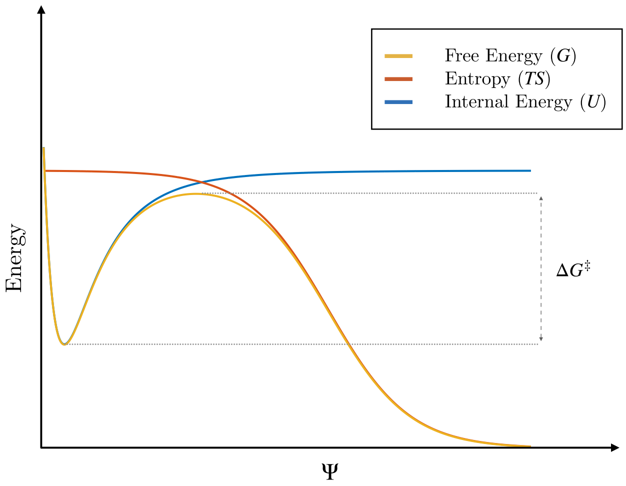

FIGURE 5 Schematic depiction of the free energy profile (*yellow curve*) associated with the rupturing process of DNA, as a function of an effective reaction coordinate Ψ. In spite of the twofold nick of the DNA backbone, non-covalent interactions hold the DNA moieties within a locally metastable thermodynamic basin. Subject to a tensile force and limited fraying of the DNA overhangs at the contact interface between the broken moieties, the local conformational freedom of DNA is restrained. A major contribution to the activation free energy of the process is thus provided by the internal energy U (*blue curve*), whereas the entropic contribution TS is approximately constant (*orange curve*). This assumption holds up to the transition state of the rupturing process, described by the activation energy $\Delta G^{\ddagger}$: in fact, as the DNA moieties drift from the lesion site, several degenerate conformations become available, associated with a significant increase in the entropy of the system. To see this figure in color, go online.

$D_{CG} = 2.2 \times 10^{-11}\ \mu m^2/\tau$ was thus inferred from a linear fitting of the mean-squared displacement versus time (see supporting material section S4). As for D_{EXP}, we relied on the experimental procedure of Robertson and co-workers (54), who estimated the diffusion coefficient of fragments of λ-phage DNA obtained by coupling the action of restriction enzymes and gel electrophoresis. We thus extrapolated the value of D_{EXP} from the experimental diffusion coefficients of longer DNA filaments, and scaled it by the ratio of the temperatures adopted in our simulations and in the experimental setup, achieving $D_{EXP} = 1.17\ \mu m^2/s$ and a time-scaling factor $\Gamma(\zeta)$ of 11.1. Results of the rescaling protocol applied to the characteristic rupture times of the DNA molecule at $\langle R_{ee} \rangle = 1000$ nm are shown in Table 4, where we artificially extended the application range of Eq. 4, extrapolating the properties of DSB motifs at larger distances ($b_d \geq 4$) in a purely speculative effort.

By implying that the rupture of DNA is a two-step process regardless of the DSB motif, driven by the abrupt disruption of the residual contact interface between the DNA moieties, we infer a wide kinetic span (10^{-8}–10^{-1} s) associated with DSB distances $b_d = 0 - 10$. Despite the limitations and lack of validation of the protocol (and several approximations thereof), our estimates are in fair agreement with the characteristic dissociation times of free DNA duplexes in solution (39)—the latter being likely characterized, however, by a non-negligible entropic contribution from the DNA fraying. In fact, a unique, earlier all-atom MD assessment on the thermal stability of DSBs over short DNA molecules reported significantly lower kinetic rates, associating the rupture of DNA by DSB motifs at $b_d = 3$ to characteristic timescales in the order of hours (22). Such a discrepancy might arise from a misestimate of the strength of non-covalent interactions, which knowingly impact classical atomistic force fields (26,55,56), but might, in principle, be expected to affect CG models alike. Yet, it is worth noting that, while the oxDNA2 force field is benchmarked upon equilibrium properties of DNA, its characterization of transient, dynamical processes has to be verified a posteriori and further validated experimentally.

CONCLUSIONS

DSBs, i.e. the twofold cut of the DNA backbone on complementary strands, are a likely and detrimental outcome of the radiation field in cell nuclei. A dynamical characterization of the local breakdown of DNA is thus critical, as it sets the stage for the subsequent cascade of events, involving the activation of the DDR machinery and the rejoining of the broken DNA moieties. In this work, we tackled the in silico assessment of the early stages of the mechanical rupture of DNA by DSBs enforced on a constrained DNA filament. We performed extensive MD simulations employing a CG model of DNA, which let us cover biologically significant timescales, yet keeping a proper level of detail of the molecular process. Moreover, we established a consistent protocol to estimate both the timescales and a characteristic internal energy contribution to the rupturing process from the diverse DSB motifs. In all scenarios, where the DNA molecule has been kept at varied (average) end-to-end distances by the application of a constant force regime, we observed an exponential increase of the average rupture times with the DSB distance, with the fitting coefficients varying negligibly upon the tensile protocol enforced. This latter observation might apply to a zero-force regime likewise, although the contributions from the enhanced entropic and diffusive terms to the kinetics of

TABLE 4 Characteristic times of the DNA rupturing associated with an external force $F_z = 0.42$ pN, rescaled *via* Eq. 8

b_d	0	1	2	3	4	5	6	7	8	9	10
$\bar{\tau}_{b_d}$ [s]	1×10^{-8}	7×10^{-8}	1×10^{-6}	2×10^{-6}	1×10^{-5}	8×10^{-5}	4×10^{-4}	2×10^{-3}	1×10^{-2}	7×10^{-2}	4×10^{-1}

Values associated with $b_d = 0 - 3$ are the (scaled) estimates shown in Table 1, whereas the characteristic rupture times relative to higher DSB distances are extrapolated employing Eq. 4.

the DNA rupturing process should be verified. We thus inferred the DNA rupture by DSBs being a thermally activated process, as corroborated by the characteristic times being distributed according to an exponential probability density (see supporting material section S5), which is associated with the likelihood of rare events. Moreover, the internal energy contribution to driving the DNA moieties from a metastable, bound state to a broken state exhibits a linear dependence on the DSB distance, suggesting that the residual base pairing holding the DNA moieties contributes to the contact energy in an additive manner within the distance range under scrutiny - namely, $b_d = 0 - 3$ - net of sequence-dependent and higher-order effects, which are worth exploring nevertheless. Overall, the rupture of DNA by DSBs is seemingly a cooperative process, most likely driven by the earliest disruption of the $\pi - \pi$ stacking interactions involving the overhanging nucleotides, from the residual contact interface between the DNA moieties. In fact, this apparent cooperativity should be verified on wider DSB motifs, arguably on conveniently shorter systems and/or at a lower-resolution level. In conclusion, in this work, we have verified the capabilities of simple and effective in silico models of DNA in describing the out-of-equilibrium phenomenology of (radiation-enforced) DNA lesions. Moreover, these simulations establish an extensive benchmark to a single-molecule experimental setup, i.e., the assessment of a thermally-activated process within an optical tweezing scenario, and an attempt at tackling the highly non-trivial scaling of time by the CG protocols. While further scrutiny is in order to properly characterize the transition path along the mechanical rupture of DNA, arguably the tools at our disposal are reliable, paving the way for further developments.

SUPPORTING MATERIAL

Supporting material can be found online at https://doi.org/10.1016/j.bpj.2023.07.008. The raw data associated with this work are freely available on a Zenodo repository at the link https://doi.org/10.5281/zenodo.7941733.

AUTHOR CONTRIBUTIONS

R.P. and G.L. oversaw and coordinated the whole research project. M.M. performed all MD simulations. The data analysis was carried out by M.M. with the constant supervision of L.P. M.M. and L.P. mainly worked on the first draft, while the final manuscript was written with contributions from all authors.

ACKNOWLEDGMENTS

The authors wish to thank Roberto Menichetti and Luca Tubiana for a critical reading and insightful comments. This project received funding from the European Research Council under the European Union's Horizon 2020 research and innovation program (grant 758588). R.P., L.P., and M.M. acknowledge support from the Italian Ministry of Education, University and Research (MIUR) through the FARE grant for the project HAMMOCK (grant R18ZHWY3NC).

DECLARATION OF INTERESTS

The authors declare no competing interests.

REFERENCES

1. Lindahl, T. 1993. Instability and decay of the primary structure of DNA. *Nature.* 362:709–715. https://doi.org/10.1038/362709a0.
2. Burrell, R. A., S. E. McClelland, ..., C. Swanton. 2013. Replication stress links structural and numerical cancer chromosomal instability. *Nature.* 494:492–496. https://doi.org/10.1038/nature11935.
3. Schipler, A., and G. Iliakis. 2013. DNA double-strand-break complexity levels and their possible contributions to the probability for error-prone processing and repair pathway choice. *Nucleic Acids Res.* 41:7589–7605. https://doi.org/10.1093/nar/gkt556.
4. Lodish, H. F., A. Berk, ..., A. Amon. 2021. Molecular Cell Biology. WH Freeman.
5. Ward, J. F. 1988. DNA damage produced by ionizing radiation in mammalian cells: Identities, mechanisms of formation, and reparability. *Prog. Nucleic Acid Res. Mol. Biol.* 35:95–125. https://doi.org/10.1016/S0079-6603(08)60611-X.
6. Goodhead, D. T. 2006. Energy deposition stochastics and track structure: What about the target? *Radiat. Prot. Dosimetry.* 122:3–15. https://doi.org/10.1093/rpd/ncl498.
7. Prise, K. M., M. Pinto, ..., B. D. Michael. 2001. A review of studies of ionizing radiation-induced double-strand break clustering. *Radiat. Res.* 156:572–576. https://doi.org/10.1667/0033-7587(2001)156[0572:AROSOI]2.0.CO;2.
8. Goodhead, D. T. 1989. The initial physical damage produced by ionizing radiations. *Int. J. Radiat. Biol.* 56:623–634. https://doi.org/10.1080/09553008914551841.
9. Barendsen, G. W. 1994. The relationships between RBE and LET for different types of lethal damage in mammalian cells: Biophysical and molecular mechanisms. *Radiat. Res.* 139:257–270. https://doi.org/10.2307/3578823.
10. McMahon, S. J. 2018. The linear quadratic model: Usage, interpretation and challenges. *Phys. Med. Biol.* 64:01TR01. https://doi.org/10.1088/1361-6560/aaf26a.
11. Rørvik, E., L. F. Fjæra, ..., K. S. Ytre-Hauge. 2018. Exploration and application of phenomenological RBE models for proton therapy. *Phys. Med. Biol.* 63, 185013. https://doi.org/10.1088/1361-6560/aad9db.
12. Hawkins, R. B. 2003. A microdosimetric-kinetic model for the effect of non-Poisson distribution of lethal lesions on the variation of RBE with LET. *Radiat. Res.* 160:61–69. https://doi.org/10.1667/RR3010.
13. Scholz, M., A. M. Kellerer, ..., G. Kraft. 1997. Computation of cell survival in heavy ion beams for therapy: The model and its approximation. *Radiat. Environ. Biophys.* 36:59–66. https://doi.org/10.1007/s004110050055.
14. Scifoni, E. 2015. Radiation biophysical aspects of charged particles: From the nanoscale to therapy. *Mod. Phys. Lett. A.* 30, 1540019. https://doi.org/10.1142/S0217732315400192.
15. Chatzipapas, K. P., P. Papadimitroulas, ..., G. C. Kagadis. 2020. Ionizing radiation and complex DNA damage: Quantifying the radiobiological damage using Monte Carlo simulations. *Cancers.* 12:799. https://doi.org/10.3390/cancers12040799.
16. Surdutovich, E., and A. V. Solov'yov. 2014. Multiscale approach to the physics of radiation damage with ions. *Eur. Phys. J. D.* 68:1–30. https://doi.org/10.1140/epjd/e2014-50004-0.
17. Nikjoo, H., P. O'Neill, ..., M. Terrissol. 1997. Computational modelling of low-energy electron-induced DNA damage by early physical and chemical events. *Int. J. Radiat. Biol.* 71:467–483. https://doi.org/10.1080/095530097143798.
18. Werner, F., M. Dingfelder, ..., P. Jacob. 2011. Track structures, DNA targets and radiation effects in the biophysical Monte Carlo simulation code PARTRAC. *Mutat. Res.* 711:28–40. https://doi.org/10.1016/j.mrfmmm.2011.01.003.

19. Lampe, N., M. Karamitros, …, S. Incerti. 2018. Mechanistic DNA damage simulations in Geant4-DNA part 1: A parameter study in a simplified geometry. *Phys. Med.* 48:135–145. https://doi.org/10.1016/j.ejmp.2018.02.011.

20. Elsässer, T., and M. Scholz. 2007. Cluster effects within the local effect model. *Radiat. Res.* 167:319–329. https://doi.org/10.1667/RR0467.1.

21. Petrolli, L., F. Tommasino, …, G. Lattanzi. 2020. Can we assess early DNA damage at the molecular scale by radiation track structure simulations? A tetranucleosome scenario in Geant4-DNA. *Front. Phys.* 8, 576284. https://doi.org/10.3389/fphy.2020.576284.

22. Landuzzi, F., P. L. Palla, and F. Cleri. 2017. Stability of radiation-damaged DNA after multiple strand breaks. *Phys. Chem. Chem. Phys.* 19:14641–14651. https://doi.org/10.1039/C7CP02266B.

23. Uusitalo, J. J., H. I. Ingólfsson, …, S. J. Marrink. 2015. Martini coarse-grained force field: Extension to DNA. *J. Chem. Theory Comput.* 11:3932–3945. https://doi.org/10.1021/acs.jctc.5b00286.

24. Freeman, G. S., D. M. Hinckley, …, J. J. de Pablo. 2014. Coarse-grained modeling of DNA curvature. *J. Chem. Phys.* 141:165103. https://doi.org/10.1063/1.4897649.

25. Ouldridge, T. E., A. A. Louis, and J. P. K. Doye. 2011. Structural, mechanical, and thermodynamic properties of a coarse-grained DNA model. *J. Chem. Phys.* 134, 085101. https://doi.org/10.1063/1.3552946.

26. Dans, P. D., J. Walther, …, M. Orozco. 2016. Multiscale simulation of DNA. *Curr. Opin. Struct. Biol.* 37:29–45. https://doi.org/10.1016/j.sbi.2015.11.011.

27. Giulini, M., M. Rigoli, …, R. Potestio. 2021. From system modeling to system analysis: The impact of resolution level and resolution distribution in the computer-aided investigation of biomolecules. *Front. Mol. Biosci.* 8, 676976. https://doi.org/10.3389/fmolb.2021.676976.

28. Ritort, F. 2006. Single-molecule experiments in biological physics: Methods and applications. *J. Phys. Condens. Matter.* 18:R531–R583. https://doi.org/10.1088/0953-8984/18/32/R01.

29. Bustamante, C. J., Y. R. Chemla, …, M. D. Wang. 2021. Optical tweezers in single-molecule biophysics. *Nat. Rev. Methods Primers.* 1:25. https://doi.org/10.1038/s43586-021-00021-6.

30. Wang, M. D., H. Yin, …, S. M. Block. 1997. Stretching DNA with optical tweezers. *Biophys. J.* 72:1335–1346. https://doi.org/10.1016/S0006-3495(97)78780-0.

31. Snodin, B. E. K., F. Randisi, …, J. P. K. Doye. 2015. Introducing improved structural properties and salt dependence into a coarse-grained model of DNA. *J. Chem. Phys.* 142:234901. https://doi.org/10.3389/fmolb.2021.645527.

32. Šulc, P., F. Romano, …, A. A. Louis. 2012. Sequence-dependent thermodynamics of a coarse-grained DNA model. *J. Chem. Phys.* 137, 135101. https://doi.org/10.1063/1.4754132.

33. Sengar, A., T. E. Ouldridge, …, P. Šulc. 2021. A primer on the oxDNA model of DNA: When to use it, how to simulate it and how to interpret the results. *Front. Mol. Biosci.* 8:693710. https://doi.org/10.3389/fmolb.2021.693710.

34. Thompson, A. P., H. M. Aktulga, …, S. J. Plimpton. 2022. LAMMPS - a flexible simulation tool for particle-based materials modeling at the atomic, meso, and continuum scales. *Comput. Phys. Commun.* 271, 108171. https://doi.org/10.1016/j.cpc.2021.108171.

35. Davidchack, R. L., T. E. Ouldridge, and M. V. Tretyakov. 2015. New Langevin and gradient thermostats for rigid body dynamics. *J. Chem. Phys.* 142, 144114. https://doi.org/10.1063/1.4916312.

36. Henrich, O., Y. A. Gutiérrez Fosado, …, T. E. Ouldridge. 2018. Coarse-grained simulation of DNA using LAMMPS. *Eur. Phys. J. E.* 41:57. https://doi.org/10.1140/epje/i2018-11669-8.

37. Bustamante, C., J. F. Marko, …, S. Smith. 1994. Entropic elasticity of λ-phage DNA. *Science.* 265:1599–1600. https://doi.org/10.1126/science.8079175.

38. Marko, J. F., and E. D. Siggia. 1995. Stretching DNA. *Macromolecules.* 28:8759–8770. https://doi.org/10.1021/ma00130a008.

39. Cocco, S., R. Monasson, and J. F. Marko. 2001. Force and kinetic barriers to unzipping of the DNA double helix. *Proc. Natl. Acad. Sci. USA.* 98:8608–8613. https://doi.org/10.1073/pnas.151257598.

40. Strunz, T., K. Oroszlan, …, H. J. Güntherodt. 1999. Dynamic force spectroscopy of single DNA molecules. *Proc. Natl. Acad. Sci. USA.* 96:11277–11282. https://doi.org/10.1073/pnas.96.20.11277.

41. Evans, E. 2001. Probing the relation between force—lifetime—and chemistry in single molecular bonds. *Annu. Rev. Biophys. Biomol. Struct.* 30:105–128. https://doi.org/10.1146/annurev.biophys.30.1.105.

42. Strick, T. R., D. Bensimon, and V. Croquette. 1999. Micro-mechanical Measurement of the Torsional Modulus of DNA. Springer, pp. 57–62. https://doi.org/10.1023/A:1003772626927.

43. Dudko, O. K., G. Hummer, and A. Szabo. 2006. Intrinsic rates and activation free energies from single-molecule pulling experiments. *Phys. Rev. Lett.* 96, 108101. https://doi.org/10.1103/PhysRevLett.96.108101.

44. Ferrario, M., G. Ciccotti, and K. Binder. 2006. *In* Computer Simulations in Condensed Matter: From Materials to Chemical Biology. Volume 1 Springer Berlin. https://doi.org/10.1007/3-540-35273-2.

45. Bolhuis, P. G., D. Chandler, …, P. L. Geissler. 2002. Transition path sampling: Throwing ropes over rough mountain passes, in the dark. *Annu. Rev. Phys. Chem.* 53:291–318. https://doi.org/10.1146/annurev.physchem.53.082301.113146.

46. Geissler, P. L., C. Dellago, and D. Chandler. 1999. Kinetic pathways of ion pair dissociation in water. *J. Phys. Chem. B.* 103:3706–3710. https://doi.org/10.1021/jp984837g.

47. Dellago, C., P. G. Bolhuis, …, D. Chandler. 1998. Transition path sampling and the calculation of rate constants. *J. Chem. Phys.* 108:1964–1977. https://doi.org/10.1063/1.475562.

48. Depa, P. K., and J. K. Maranas. 2005. Speed up of dynamic observables in coarse-grained molecular-dynamics simulations of unentangled polymers. *J. Chem. Phys.* 123, 094901. https://doi.org/10.1063/1.1997150.

49. Accary, J. B., and V. Teboul. 2012. Time versus temperature rescaling for coarse grain molecular dynamics simulations. *J. Chem. Phys.* 136, 094502. https://doi.org/10.1063/1.3690094.

50. Ouldridge, T. E. 2012. Coarse-grained Modelling of DNA and DNA Self-Assembly. Springer Berlin.

51. Doi, M., and S. F. Edwards. 1988. The Theory of Polymer Dynamics73. Oxford University Press.

52. Clausen-Schaumann, H., M. Rief, …, H. E. Gaub. 2000. Mechanical stability of single DNA molecules. *Biophys. J.* 78:1997–2007. https://doi.org/10.1016/S0006-3495(00)76747-6.

53. Ouldridge, T. E., P. Sulc, …, A. A. Louis. 2013. DNA hybridization kinetics: zippering, internal displacement and sequence dependence. *Nucleic Acids Res.* 41:8886–8895. https://doi.org/10.1093/nar/gkt687.

54. Robertson, R. M., S. Laib, and D. E. Smith. 2006. Diffusion of isolated DNA molecules: dependence on length and topology. *Proc. Natl. Acad. Sci. USA.* 103:7310–7314. https://doi.org/10.1073/pnas.0601903103.

55. Yoo, J., D. Winogradoff, and A. Aksimentiev. 2020. Molecular dynamics simulations of DNA-DNA and DNA-protein interactions. *Curr. Opin. Struct. Biol.* 64:88–96. https://doi.org/10.1016/j.sbi.2020.06.007.

56. Nerenberg, P. S., and T. Head-Gordon. 2018. New developments in force fields for biomolecular simulations. *Curr. Opin. Struct. Biol.* 49:129–138. https://doi.org/10.1016/j.sbi.2018.02.002.

Biophysical Journal, Volume *122*

Supplemental information

Kinetics of radiation-induced DNA double-strand breaks through coarse-grained simulations

Manuel Micheloni, Lorenzo Petrolli, Gianluca Lattanzi, and Raffaello Potestio

Supplementary Material for *Kinetics of radiation-induced DNA double-strand breaks through coarse-grained simulations*

Manuel Micheloni[a,b], Lorenzo Petrolli[a,b], Gianluca Lattanzi[a,b] and Raffaello Potestio[a,b,*]

[a] *Physics Department, University of Trento, Trento, Italy*
[b] *INFN-TIFPA, Trento Institute for Fundamental Physics and Applications, Trento, Italy*

1 DNA sequence template

Here, we report the DNA sequence template employed to construct the double-stranded DNA molecule, while Fig. S1 shows the local nucleotide sequence associated with each DSB motif.

```
   1 ATGGTTTATT CTTATACTGA AAAAAAACGT ATTCGTAAAG ATTTTGGTAA ACGTCCTCAA GTTTTAGATG
  71 TTCCTTATTT ATTATCTATT CAATTAGATT CTTTTCAAAA ATTTATTGAA CAAGATCCTG AAGGTCAATA
 141 TGGTTTAGAA TTTCGTTCTG TTTTTCCTAT TCAATCCTAT TCCGGTAATT CCGAATTGCA ATATGTTTCC
 211 TATCGCTTGG GTGAACCTGT TTTTGATGTT CAAGAATGTC AAATTCGCGG TGTTACTTAT TCCCCTTTGC
 281 GCGTTAAATT GCGCTTGGTT ATTTATGAAC GCGAACCTGA AGGTACTGTT AAAGATATTA AAGAACAAGA
 351 AGTTTATATG GGTGAAATTC CTTTAATGAC TGATAATGGT ACTTTTGTTA TTAATGGTAC TGAACGTGTT
 421 ATTGTTTCTC AATTACATCG TTCTCCTGGT GTTTTTTTTG ATTCTGATAA AGGTAAAACT CATTCTTCTG
 491 GTAAAGTTTT ATATAATCGT ATTATTCCTT ATCGTGGTTC TTGGTTAGAT TTTGAATTTG ATCCTAAAGA
 561 TAATTTATTT GTTCGTATTG ATCGTCGTCG TAAATTACCT ACTATTATTT TACGTTTAAA TTATACTACT
 631 GAACAAATTT TAGATTTATT TTTTGAAAAA GTTATTTTTG AAATTCGTGA TAATAAATTA CAAATGGAAT
 701 TGGTCCCCGA ACGCTTGCGC GGCGAAACCT CCTTTGATAT TGAAAATGGC AAAGTCTATG TCGAAAAAGG
 771 CCGCCGCATT ACCCGCCATA TTCGCCAATT GGAAAAAGAT GATGTCAAAT TGATTGAAGT CCCCGTCGAA
 841 TATATTGGCA AAGTCGTCAA AGATTATATT GATGAATCCA CTGGTGAATT GATTTGTAAT ATGGAATTGT
 911 CCTTGGATTT GTTGAAATTG TCCCAATCCG GTCATAAACG CATTGAAACT TTGTTTACTA ATGATTTGGA
 981 TCATGGTCCT TATATTTCCG AAACTTTGCG CGTTGATCCT ACTAATGATC GCTTGTCGCT CGTAGAGATC
1051 TACCGGATGA TGCGGCCAGG AGAGCCACCA ACACGGGAGG AGTCGCTCTT CGAGAACCTC TTCTTCTCGG
1121 AGGACCGGTA CGACCTCTCG GTAGGACGGA TGAAGTTCAA CCGGTCGCTC CTCCGGGAGG AGATCGAGGG
1191 ATCGGGAATC CTCTCCAAAG ATGATATTAT TGATGTTATG AAAAAATTGA TTGATATTCG CAATGGTAAA
1261 GGTGAAGTTG ATGATATTGA TCATTTGGGT AATCGCCGCA TTCGCTCCGT TGGTGAAATG GAAAATCAAT
1331 TTCGCGTTGG TTTGGTTCGC GTTGAACGCG TTAAAGAACG CTTGTCCCTG GGGGACCTGG ACACGCTGAT
1401 GCCGCAGGAC ATGATAAACA AGCCGATAAG CGTGAAGGAG TTCTTCGGGA GCAGCCAGCT GAGCCAGTTC
1471 ATGGACCAGA ACAACCCGCT GAGCGAGATA ACGCACAAGA GGAGGATAAG CCTGGGGCCG GGGGGGCTGA
1541 CGAGGGAGAG GGGGTTCGAG GTGAGGGACG TGCACCCGAC GCACTACGGG AGGGTGTGAC CGATAGAGAC
1611 GCCGGAGGGG CCGAACATAG GGCTGATAAA CAGCCTGAGC GTGTACCAGA CGAACGAGTA CGGGTTCCTG
```

* raffaello.potestio@unitn.it

1681 GAGACGCCGT ACAGGAAGGT GACGGACGGG GTGGTGACGG ACGAGATACA CTACCTGAGC ATAGAGGAGG
1751 GGAACTACGT GATACAGAAC AGCAACCTGG ACGAGGAGGG GCACTTCGTG GAGGACCTGG TGACGTGAAG
1821 GAGCAAGGGG GAGAGCAGCC TGTTCAGCAG GGACCAGGTG GACTACATGG ACGTGAGCAC GCAGCAGGTG
1891 GTTTCCGTTG GTTCCTTGAT TCCTTTTTTG GAACATGATG ATAATCGCTT GATGGGTAAT ATGCAACGCC
1961 AAGTTCCTAC TTTGCGCGAT AAACCTTTGG TTGGTACTGG TATGGAACGC GTTGTTGATT CCGGTGTTAC
2031 TGTTAAACGC GGTGGTGTTG TTCAATATGT TGATTCCCGC ATTGTTATTA AAGTTAATGA AGATGAAATG
2101 TATCCTGGTG AAGGTATTGA TATTTATAAT TTGACTAAAT ATACTCGCTC CAATCAAAAT ACTTGTATTA
2171 ATCAAATGCC TTGTGTTTCC TTGGGTGAAC CTGTTGAACG CGGTGATGTC TTGGATGGCC CCTCCACCGA
2241 TTTGGGCGAA TTGTTGGGCC AAAATATGCG CGTCTTTATG CCCTGGAATG GCTATAATTT TGAAGATTCC
2311 ATTTTGGTCT CCGAACGCGT CGTCCAAGAA GATCGCTTTA CCACCATTCA TATTCAAGAA TTGTGTGTCT
2381 CCCGTGATAC TAAATTAGGT CCTGAAGAAA TTACTGATAT TCCTAATGTT GGTGAATTAT CTAAATTAGA
2451 TGAATCTGGT ATTGTTTATA TTGGTGAAGT TACTGGTGGT GATATTTTAG TTGGTAAAGT TACTCCTAAA
2521 GGTGAAACTC AATTAACTCC TGAAGAAAAA TTATTACGTA TTTTTGGTGA AAAATCTGAT GTTAAAGATT
2591 CTTCTTTACG TGTTCCTAAT GGTGTTTCTG GTACTGTTAT TGATGTTCAA GTTTTTACTC GTGATGGTGT
2661 TGAAAAAGAT AAACGTTTAG AAATTGAAGA AATGCAATTA AAACAAAAAA AAGATTTATC GGAAGAACTC
2731 CAAATCCTCG AAGGCCTCTT TTCGCGGATC CGGGTCCTCG TCGGCGGCGT CGAAGAAAAA CTCGATAAAC
2801 TCCCCCGGGA TCGGTGGCTC GAACTCGGCC TCACCGATGA AGAAAAACAA AATCAACTCG AACAACTCGA
2871 ACAATATGAT GAACTCAAGC ACGAGTTCGA GAAGAAGCTC GAGAAGCGGC GGAAGATCAC ACAGGGAGAC
2941 GACCTCCCAG GAGTACTCAA GATCGTAAAG GTATACCTCG TAAAGCGGCG GATCCAGCCA GGAGACAAGA
3011 TGGGACGGCA CGGAAACAAG GGAGTAATCT CGAAGATCAA CCCAATCGAG GACATGCCAT ACGACGAGAA
3081 CGGAACACCA GTAGACATCG TACTCAACCC ACTCGGAGTA CCATCGCGGA TGAACATCGG ACAGATCCTC
3151 GAGACACACC TCGGAATGAA GGGAATCGGA GACAAGATCA ACATGCTCAA GCAGCAGCAG GAGGTAAAGC
3221 TAAGAGAGTT CATACAGAGA TACGACCTAG GAGACGTAAG ACAGAAGGTA GACCTAAGTA CATTCAGTGA
3291 CGAGGAGGTA ATGAGACTAG AGAACCTAAG AAAGGGAATG CCAATAACAC CAGTATTCGA CGGAAAGGAG
3361 GAGATAAAGG AGCTACTAAA GCTGGGGGAC CTGCCGACGA GCGGGCAGAT AAGGCTGTAC GACGGGAGGA
3431 CGGGGGAGCA GTTCGAGAGG CCGGTGACGG TGGGGTACAT GTACATGCTG AAGCTGAACC ACCTGGTGGA
3501 CGACAAGATG CACAGGAGCA CGGGGAGCTA CAGCCTGGTG ACGCAGCAGC CGCTGGGGGG TAAACAATTT
3571 GGTGGTCAAC GTTTTGGTGA AATGGAAGTT TGGTTAGAAT ATGGTTATAC TTTACAAGAA ATGTTAACTG
3641 TTAAATCTGA TGATGTTAAT GGTCGTACTA AAATGTATAA AAATATTGTT GATGGTAATC ATCAAATGGA
3711 ACCTGGTATG CCTGAGAGCT TCAACGTGCT GCTGAAGGAG ATAAGGAGCC TGGGGATAAA CATAGAGCTG
3781 GAGGACGAGG AGAGCTTCAA CGTGCTGCTG AAGGAGATAA GGAGCCTGGG GATAAACATA GAGCTGGAGG
3851 ACGAG

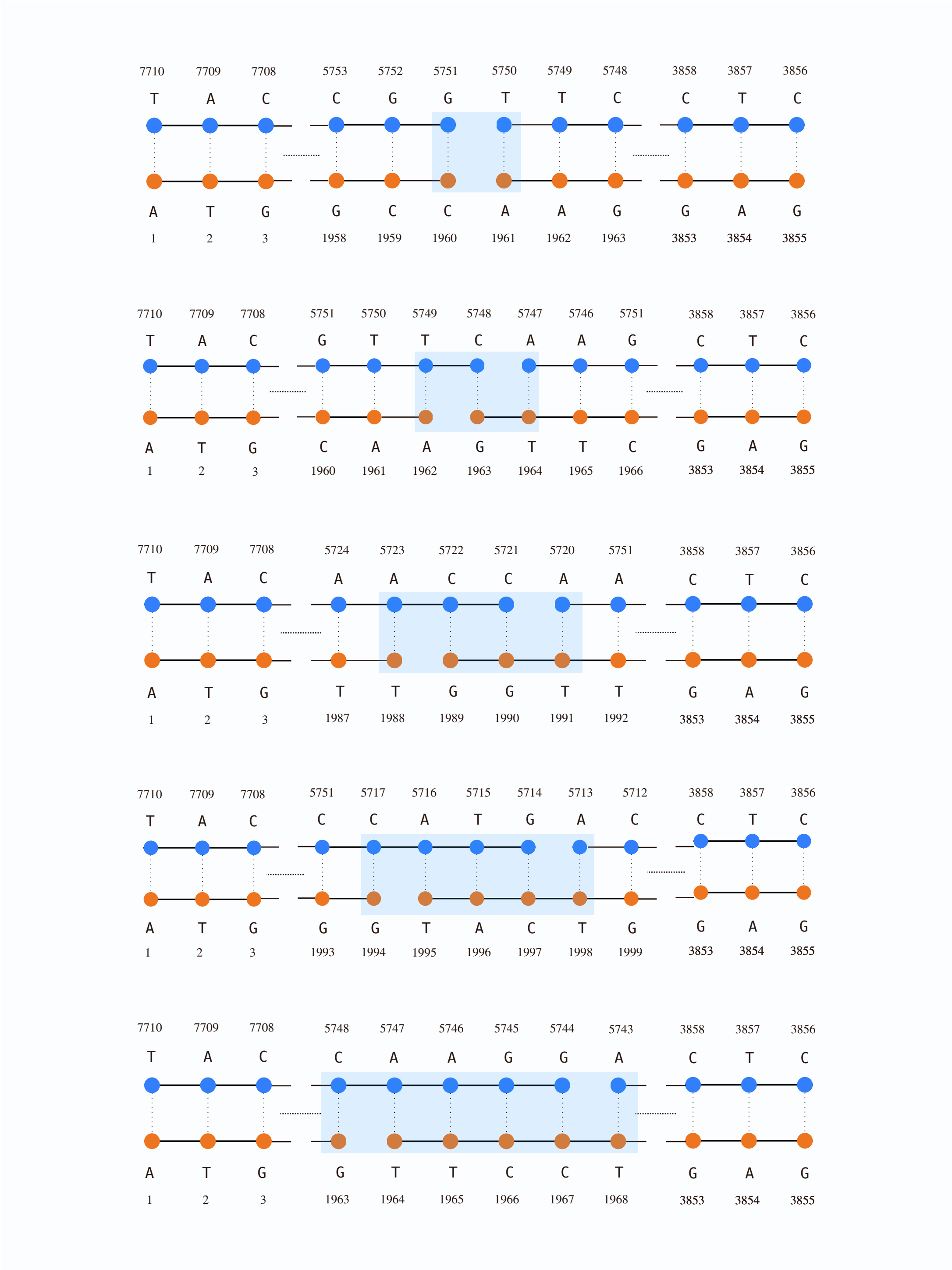

Supplementary Figure S1: Schematic depiction of the DSB motifs. Nucleotides are shown as circles; covalent bonds of the DNA backbone as solid lines; Watson-Crick hydrogen bonds between the DNA strands as dotted lines. To enforce a DSB, covalent bonds are removed between adjacent nucleotides from both DNA strands. Each nucleotide is associated with the residue type (A, T, C, G) and the respective LAMMPS atom-ID.

2 Molecular dynamics simulations: DNA thermalization protocol

We carried out a step-wise thermalization protocol as follows: i) we let the DNA molecule relax at $T = 273$ K upon reaching a plateau of the total energy of the system; ii) we heated the system to a working temperature of $T = 310$ K in a quasi-static manner, by performing a single run of $1.6 \times 10^5\ \tau$ in the NVT ensemble, associated with a damping coefficient of the Langevin thermostat $\zeta = 10\ \tau$; iii) we finally ran a subsequent simulation of $3.3 \times 10^5\ \tau$ employing an underdamped Langevin thermostat ($\zeta = 100\ \tau$), to relax the molecule into an equilibrated conformation.
We carried out several instances of steered MD, relaxing the DNA molecule at different elongations (or tensile protocols) - namely, $R_{ee} = 1000,\ 1100,\ 1200$ nm, with R_{ee} the end-to-end distance of the DNA chain [6].
Such conformations were obtained by employing soft harmonic potentials restraining both DNA termini, associated with elastic constants $k_1 = 5.7$ N/m and $k_2 = 2.3 \times 10^{-3}$ N/m respectively, at $\zeta = 10\ \tau$. This protocol allowed us to slowly stretch the thermalized DNA molecule to each of the target elongations. Upon reaching the desired DNA conformation, we thus performed subsequent equilibration stages of i) $4.9 \times 10^4\ \tau$ at $\zeta = 100\ \tau$, and ii) $5.8 \times 10^5\ \tau$ at $\zeta = 10\ \tau$. The three configurations of the DNA helix served as initial frames of a set of classic MD runs aimed at assessing the early non-equilibrium effects of DSBs.

3 Heuristic assessment of the rupture of DNA by a DSB

Double-strand breaks (DSBs) are enforced on a DNA molecule by removing the covalent bonds between adjacent nucleotides over complementary strands - that is, between (n_i, n_{i+1}) and between (n_j, n_{j+1}). To devise a consistent criterion that unequivocally characterizes the rupture of DNA, we ascertain the disruption of all residual contacts between the DNA moieties by satisfying the following relation:

$$\min\{r_{i,i+N+1}, r_{j,j+N+1}\} > 2 \times b_d \langle r_{\mathrm{B}} \rangle + \Lambda, \tag{1}$$

where $r_{i,i+N+1}$ and $r_{j,j+N+1}$ are distances between indexed nucleotides (shown in Fig. S2), and $\langle r_{\mathrm{B}} \rangle$ is the equilibrium distance between adjacent nucleotides from a 2.5×10^6 τ, NVT simulation of the intact 3855-bp DNA molecule held at an end-to-end distance of 1000 nm. Λ is defined heuristically *via* a recursive protocol accounting for all MD trajectories, thus fulfilling a consistent criterion across all DSB motifs (as shown in Fig. S3), and has been set to 1.7 nm.

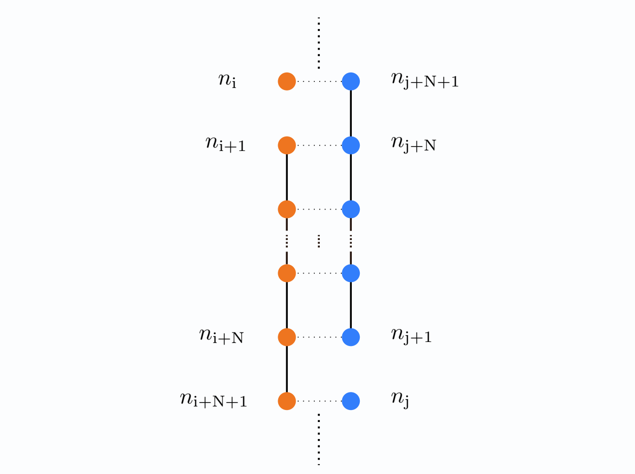

Supplementary Figure S2: Schematic depiction of a DSB motif at an arbitrary distance $b_d = \mathrm{N}$. Nucleotides are shown as circles; covalent bonds of the DNA backbone as solid lines; Watson-Crick hydrogen bonds between the DNA strands as dotted lines. To enforce a DSB, covalent bonds are removed between adjacent nucleotides from both DNA strands (between (n_i, n_{i+1}) and (n_j, n_{j+1})).

3.1 The fitting protocol

To assess the characteristic times of the DNA rupturing process τ_{b_d} from all independent simulations, we fitted the data from the variation of internal energy at the residual contact interface between the broken DNA moieties by a step-wise protocol as follows:

- we first employed Eq. 1 to approximately estimate $\tilde{\tau}_{b_d}$, thereby defining a time interval $[t_{\min}, t_{\mathrm{end}}]$, such that $\tilde{\tau}_{b_d} \in [t_{\min}, t_{\mathrm{end}}]$. The range is set to optimize the time frame to fit the internal energy profile;
- to further improve the quality of the fitting, we refined the dataset by performing a moving average of the internal energy contribution within the $[t_{\min}, t_{\mathrm{end}}]$ frame;
- we thus exploited the **sigm_fit** modulus of Matlab® to fit the internal energy profile U/k_BT by a sigmoidal function of the form:

$$E_{\mathrm{fit}} = E_{\min} + \frac{E_{\max} - E_{\min}}{1 + 10^{m(t_{50} - t)}}, \tag{2}$$

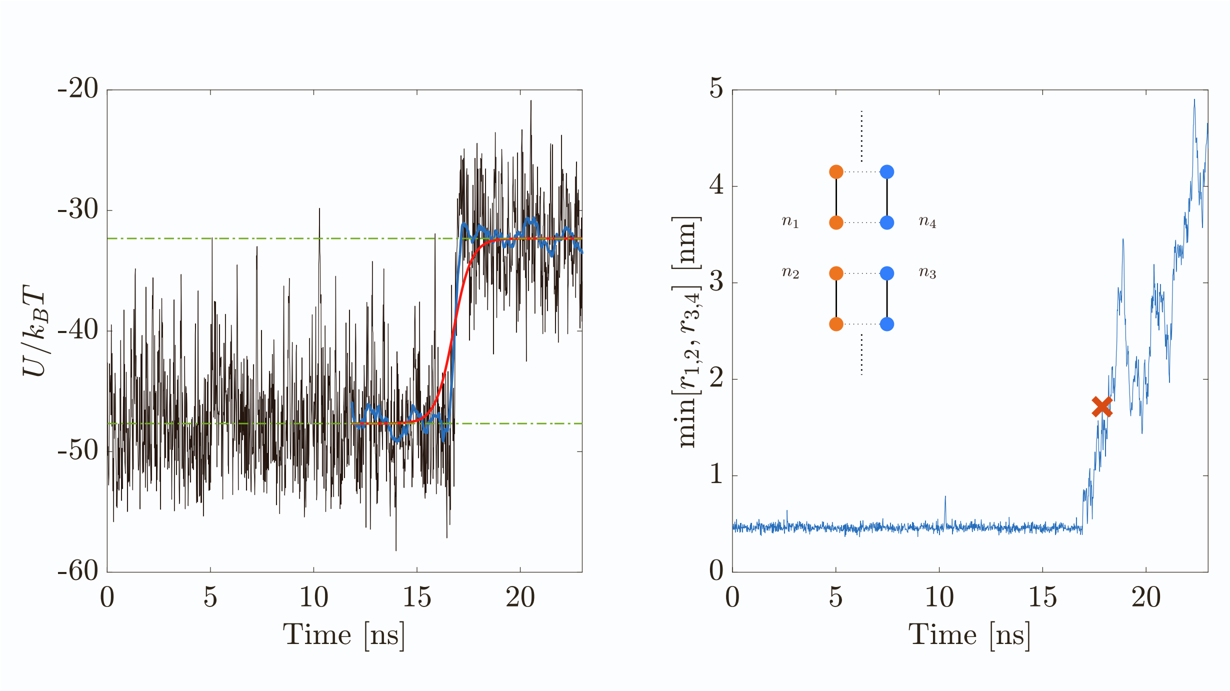

Supplementary Figure S3: **Left:** The internal energy contribution associated with the rupture of DNA from a DSB motif at distance $b_d = 0$, enforced on a 3855-bp DNA molecule experiencing an external force $F_z = 0.42$ pN. The moving average of the internal energy (shown in blue) is fitted by a sigmoid curve (Eq. 2 - red line). Dashed green lines highlight the internal energy plateaus of the (metastable) bound and broken states of the DNA molecule. **Right:** Time evolution of the heuristic distance criterion, as described by Eq. 1, associated with the rupture of DNA from a blunt DSB motif, i.e. at distance $b_d = 0$ (details in the text) - the threshold set by Eq. 1 is highlighted by a red mark.

where $E_{\rm min}$ and $E_{\rm max}$ are associated with the lower and upper plateau of the internal energy respectively, m defines the steepness of the curve, and $t_{50} = \tau_{b_d}$ is estimated at the flex of the internal energy transition.

For each DSB scenario, this protocol thus provides us with a characteristic time of the DNA rupturing process τ_{b_d} (or t_{50}), and an estimate of the internal energy of the (metastable) bound and broken states as $E_{\rm min}$ and $E_{\rm max}$ respectively. Moreover, we consistently obtain an internal energy contribution to the rupturing process from the sharp transition of U/k_BT as:

$$\Delta U/k_BT = E_{\rm fit}(\tau_{b_d}) - E_{\rm min}. \tag{3}$$

Further details on the scripts are available at the Zenodo repository. Lastly, we remark that the estimates of the rupture times from the heuristic criterion (Eq. 1) match those extrapolated from the sigmoidal fitting procedure, thereby validating the fitting protocol.

4 Derivation of the CG diffusion coefficient of freely-diffusing DNA

To extract the diffusion coefficient of the 3855-bp DNA molecule in the CG model, we started from a thermalized conformation of the 1000-nm steered DNA molecule and allowed it to diffuse freely. We thus performed a MD simulation of $5 \times 10^6\ \tau$ in the NVT ensemble, employing a damping coefficient of the Langevin thermostat $\zeta = 2.5\ \tau$, thereby extracting $D_{\mathrm{CG}}(\zeta)$ from the linear fitting of the the mean-squared displacement (MSD) profile (shown in Fig. S4).

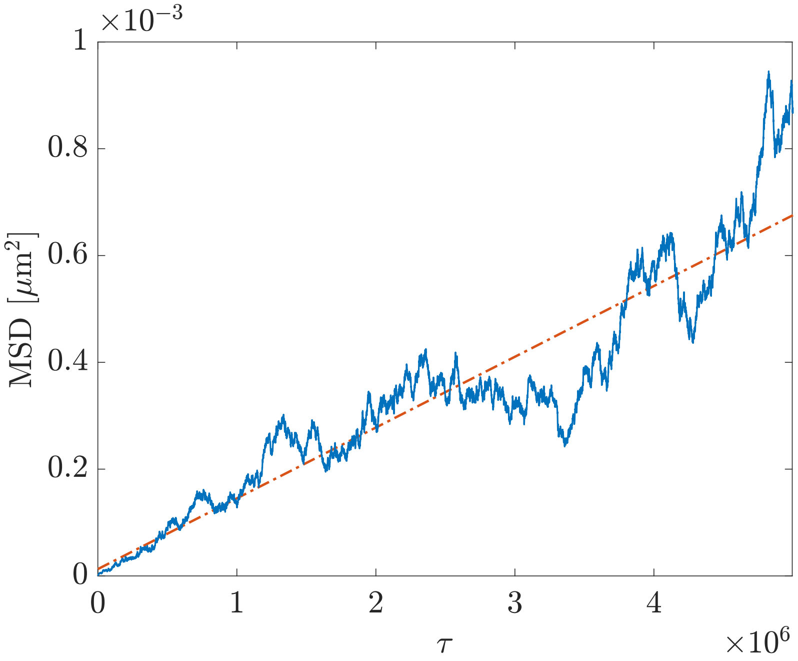

Supplementary Figure S4: Linear fitting of the MSD for the freely-diffusing, 3855-bp DNA molecule as a function of time, employing a damping coefficient of the Langevin thermostat $\zeta = 2.5\ \tau$.

5 Statistical analysis of the rupturing events

According to the data shown in Fig. S5, reporting the distribution of the rupturing times for each DSB scenario, we might characterize the rupture of DNA by a DSB as a rare event, associated with a Poisson process. The probability $P_\lambda[t]$ for the DNA molecule to break within an arbitrary time t is defined as:

$$P_\lambda[t] = 1 - \exp\left[-\lambda(b_d)t\right] \tag{4}$$

with λ^{-1} the average, characteristic time for the rupturing of DNA by a DSB motif (b_d). Thus, the probability density function of the underlying process is exponential.
To verify the reliability of our dataset, we sampled the cumulative distribution function, employing:

$$\frac{N(\tau_{b_d})}{N_0} = 1 - \exp\left[-\lambda\tau_{b_d}\right] \tag{5}$$

with $N(\tau_{b_d})$ the amount of effective simulations detecting the rupture of DNA by time τ_{b_d}, and N_0 the total amount of independent MD simulations for a (b_d, F_z) scenario, i.e. either showing a rupture event or hitting wall-time first (maximum threshold of 10^8 MD steps). Therefore, we performed a fitting procedure over Eq. 5 for each DSB scenario (Fig. S6), thereby extracting the values of λ^{-1}.

F_z [pN]	$b_d = 0$		$b_d = 1$		$b_d = 2$		$b_d = 3$	
	λ^{-1} [ns]	$\langle . \rangle$ [ns]	λ^{-1} [ns]	$\langle . \rangle$ [ns]	λ^{-1} [ns]	$\langle . \rangle$ [ns]	λ^{-1} [ns]	$\langle . \rangle$ [ns]
0.42	1	1 (488)	7	6 (477)	103	105 (122)	145	139 (106)
0.88	0.8	0.8 (427)	4	4 (504)	93	105 (102)	146	138 (77)
3.06	0.7	0.9 (478)	6	6 (525)	86	88 (108)	119	114 (94)

Supplementary Table S1: Comparison between the estimates of the average rupture times from the fitting procedure based on Eq. 5 (λ^{-1}) and from the MD simulations. The total amount of simulations performed for each scenario is reported in brackets.

As reported in Table S1, the estimates of the characteristic rupture times provided by the fitting procedure match those obtained from the average of the MD simulations.

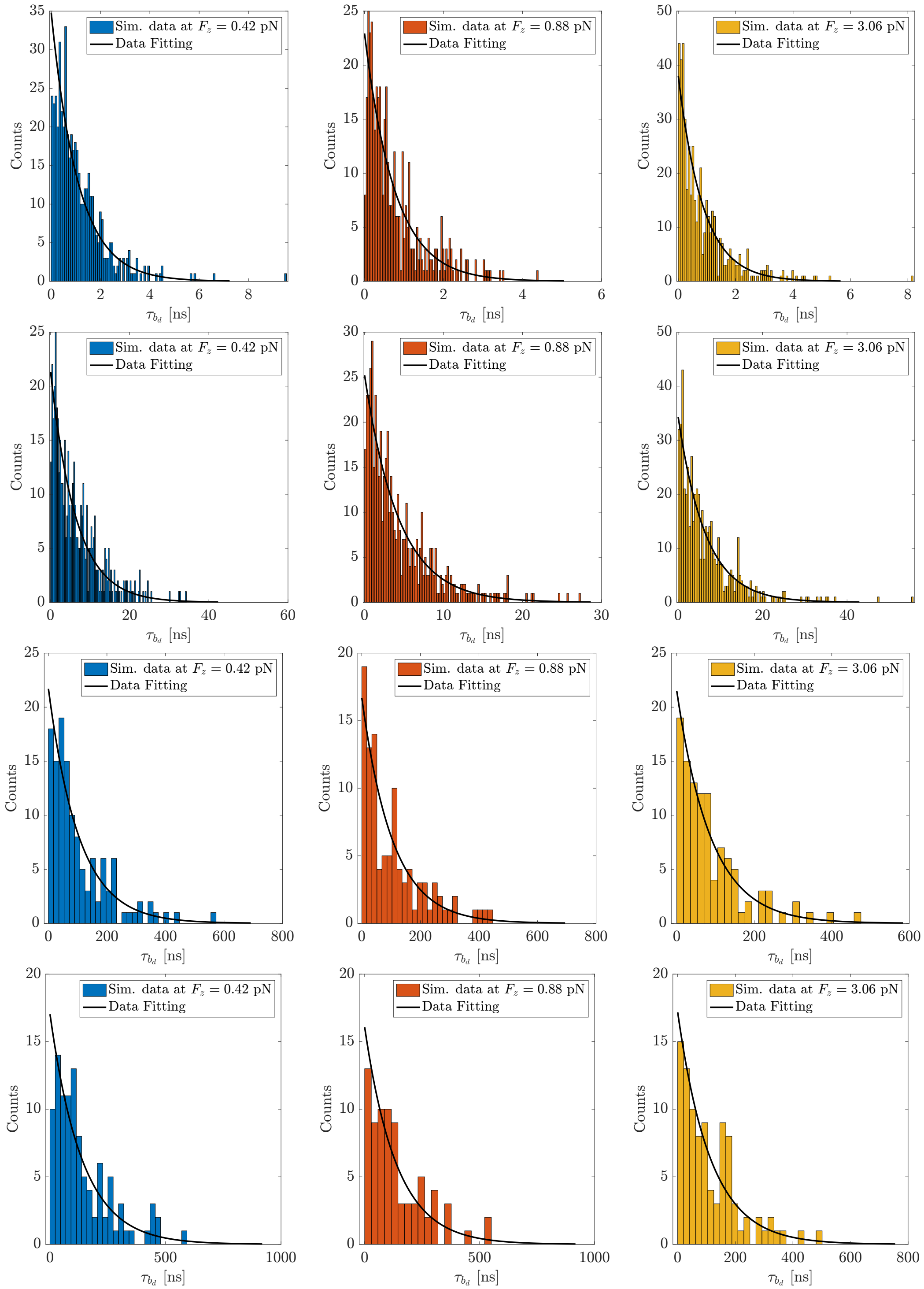

Supplementary Figure S5: Histograms of the rupturing times τ_{b_d}, showing the underlying exponential probability density function for different DSB scenarios. Each row is associated with the diverse DSB motifs ($b_d = 0 \div 3$ from top to bottom). The solid black lines highlights the fitting procedure over the corresponding dataset.

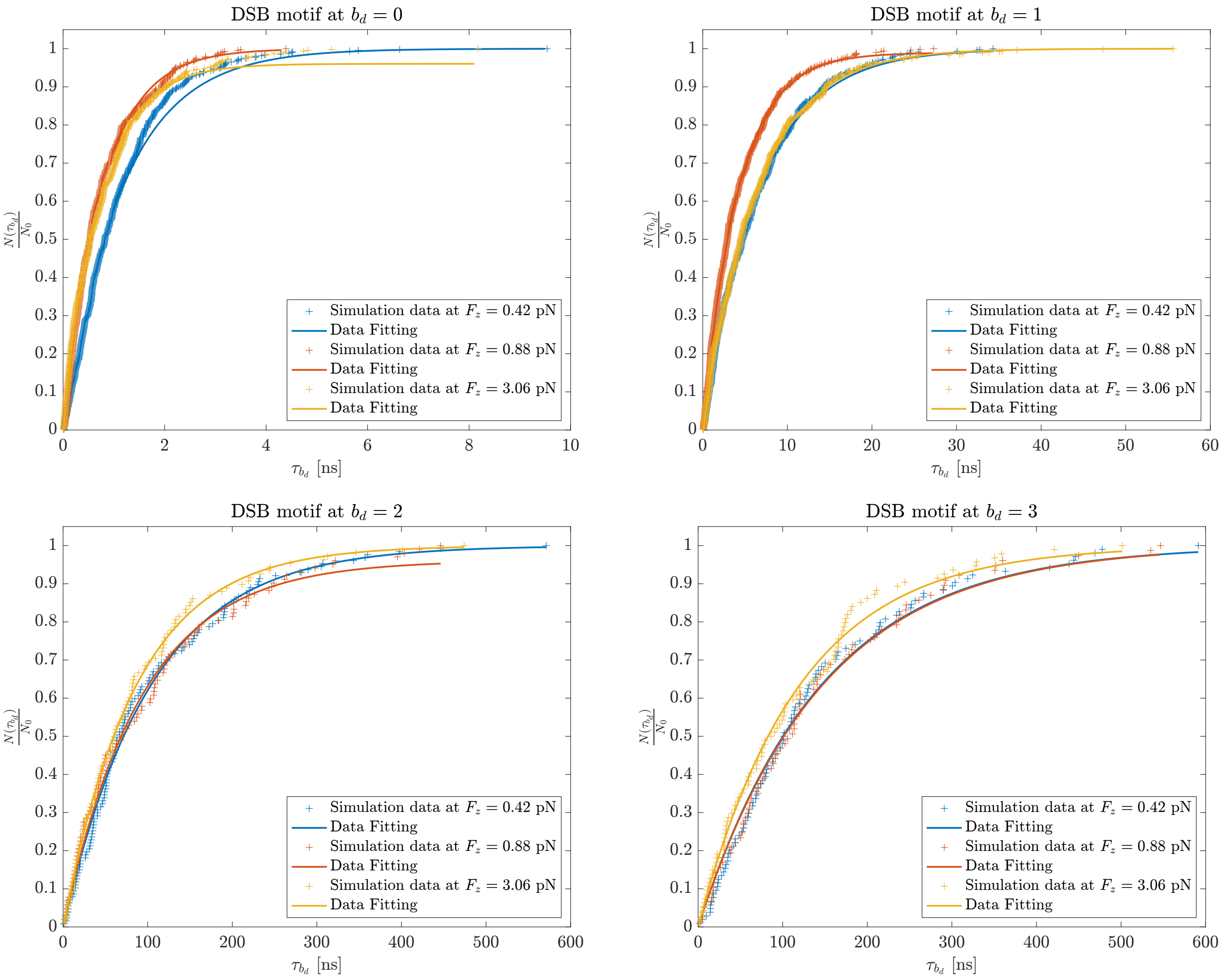

Supplementary Figure S6: Cumulative distribution function (as described by Eq. 5) for the different DSB motifs and scenarios - the solid lines show the results from the fitting procedure on the rupture times τ_{b_d} extracted from the MD simulations.

6 Analysis of the forces acting on the system

It is well-acknowledged that the application of an external force generally lowers the free energy barrier along the reaction coordinate of an activated process [2, 3, 4]. However, one might expect that a steering protocol acting in the low-force regime (that is $F_z \leq 5$ pN for the system at hand) should induce conformational changes according to the entropic compliance of the DNA molecule, rather than to an enthalpic, elastic response [1, 6, 5].
To validate the hypothesis that the simulation protocol has a negligible impact on the characteristic rupture times (as well as on the activation energy of the process), thereby allowing the DNA rupturing by thermal fluctuations, we characterized the force profile acting on the DNA molecule.

6.1 Force fluctuations

We performed about 7.5 μs-long plain MD simulations of intact DNA molecules subject to $F_z = 0.42, 0.88$ and 3.06 pN. For each trajectory, we computed the fluctuations of the total force acting on each DNA centroid, $\sigma^2[|\vec{F}_{tot}|]$, and compared the results against the force contributions from the thermostat alone - the latter defined by the fluctuation-dissipation theorem:

$$\langle F_{tot}^2 \rangle = \frac{6k_B T m}{\tau_{in} dt}, \tag{6}$$

with F_{tot} the total force on a DNA centroid, k_B the Boltzmann constant, T the temperature, m the mass of the nucleotide and τ_{in} the damping coefficient of the thermostat. As shown in Fig. S7, the fluctuations from the two contributions are of the same order of magnitude.

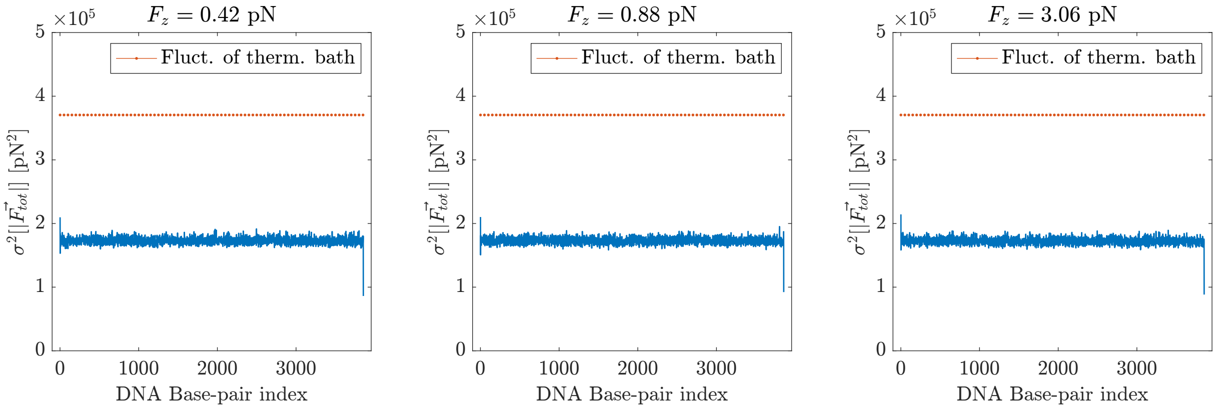

Supplementary Figure S7: Fluctuations of the forces acting on each base pair (blue line), against the sole contribution of the Langevin thermostat (orange dashed line), the latter estimated by the fluctuation-dissipation theorem.

6.2 Force correlations along the DNA molecule

We then assessed the propagation of the forces associated with the external force F_z along the DNA molecules, which might eventually enhance the kinetics of the DNA rupture.
Firstly, we performed a 'point-wise' correlation analysis of the forces acting on the centroid of the DNA terminus (i.e. experiencing F_z) and on several centroids at various distances along the DNA

molecule. The correlation (normalized by the standard deviations of the respective contributions) is defined as:

$$C_{ij}(\tau) = \frac{\langle F_z(i) F_z(j) \rangle}{\sigma[F_z(i)]\sigma[F_z(j)]} = \frac{1}{\sigma[F_z(i)]\sigma[F_z(j)]} \frac{1}{\mathcal{T} - \tau} \sum_{t=0}^{\mathcal{T}-\tau} F_z^i(t) F_z^j(t+\tau), \tag{7}$$

with i and j indexes of the DNA centroids along the chain and $\mathcal{T}$ the total simulation time. Results are depicted in Fig. S8 for the DNA molecule experiencing an external force of $F_z = 3.06$ pN - similar trends are obtained for $F_z = 0.42, 0.88$ pN, where: i) C_1 is the auto-correlation of the forces acting on the terminal nucleotides; ii) C_2 is the correlation between the forces acting on the terminal nucleotides and the forces acting on nucleotides lying at 150 bp (i.e. the DNA persistence length); iii) C_3 is the correlation between the forces acting on the terminal nucleotides and the forces acting on nucleotides lying at 2000 bp (i.e. about the lesion site). Arguably, force correlations are quickly suppressed within a few base pairs.

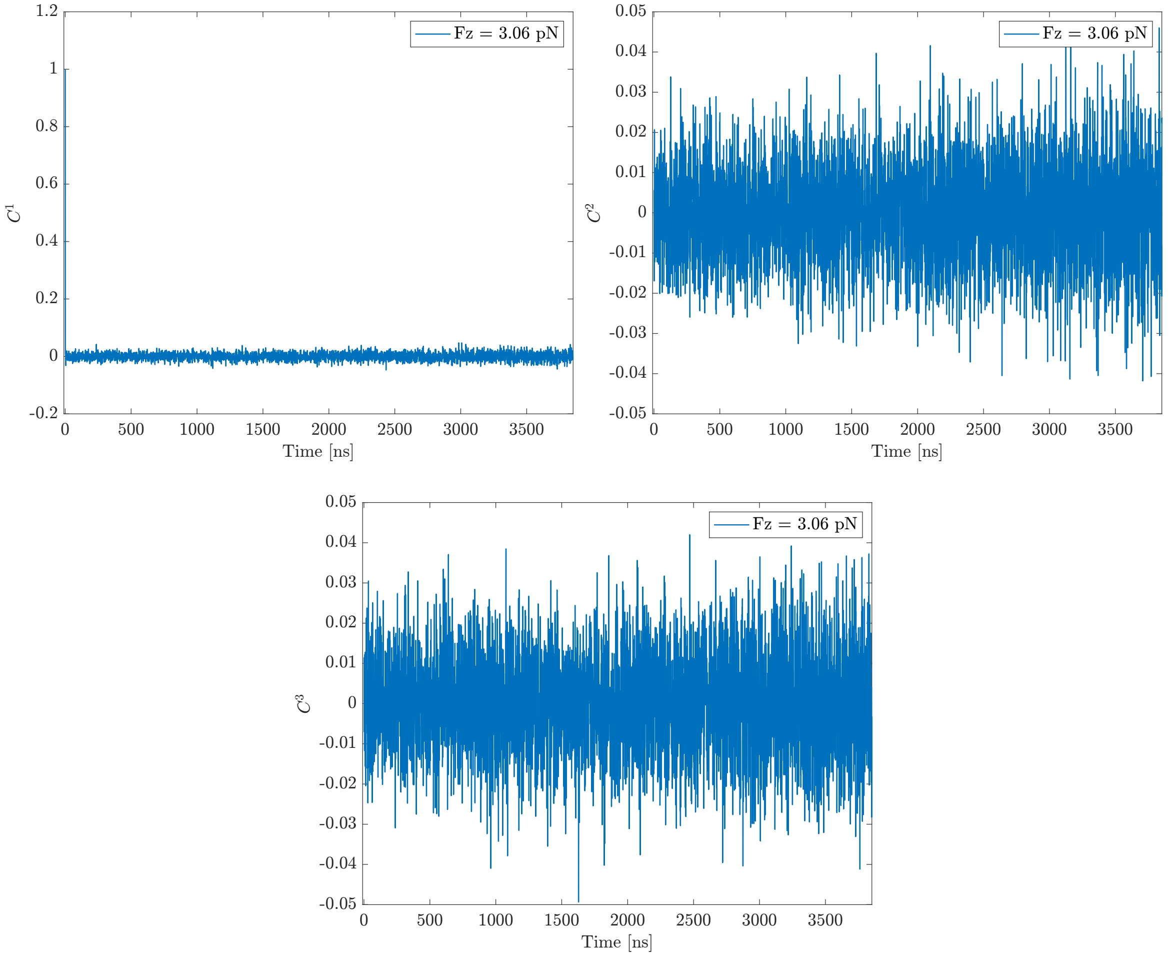

Supplementary Figure S8: Top-left: the auto-correlation of the forces acting on the terminal nucleotides (i.e. experiencing an external force of $F_z = 3.06$ pN). **Top-right**, **bottom**: correlation between the forces acting on the terminal nucleotides and on the nucleotides lying at 150 and 2000 bp from the DNA terminus, respectively.

Subsequently, we employed Eq. 7 to derive the force correlations between DNA centroids at distance

s anywhere along the chain:

$$\langle C(\tau) \rangle_s = \frac{1}{N(s)} \sum_s C_{s=|i-j|}(\tau) \tag{8}$$

with $N(s)$ the amount of unique (i, j) correlations between nucleotides at distance $s = |i - j|$. Likewise, results are shown in Fig. S9 and report a quickly-decaying correlation of the forces along the DNA molecule.

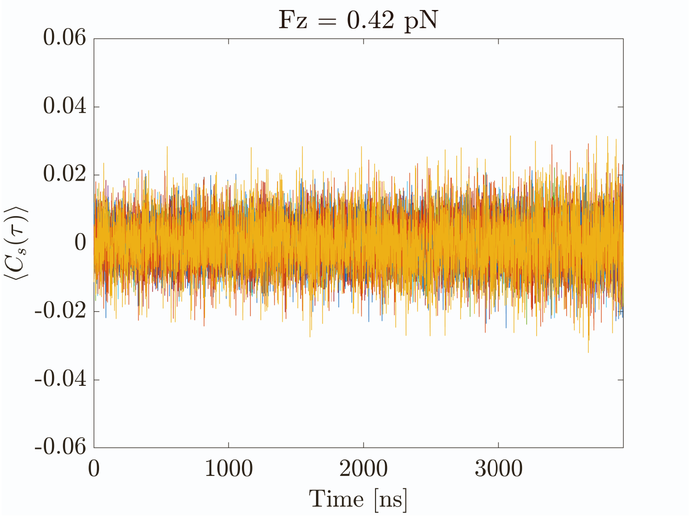

Supplementary Figure S9: Average correlations of the forces acting on nucleotides lying at distance $s = 1, 2, ..., 3855$ bp along the DNA chain - tracks are laid on top of one another.

7 DNA rupture kinetics and zero-force extrapolation

To further corroborate the hypothesis that the application of an external force in the low-force regime is associated with a negligible effect on the kinetics of the DNA rupture - so that the process is mostly driven by thermal fluctuations, Fig. S10 shows a (roughly) constant trend of the characteristic rupture times $\bar{\tau}_{b_d}$ as function of F_z. By single molecule experiments, Cocco and co-workers showed that the dissociation times associated with the unzipping of DNA helices is a quadratic function of the force in the low-force regime [2]. From Fig. S10, we appreciate a slight decrease of the rupture times versus the applied force at $b_d = 2$ and 3 - which is, however, associated with a large uncertainty.

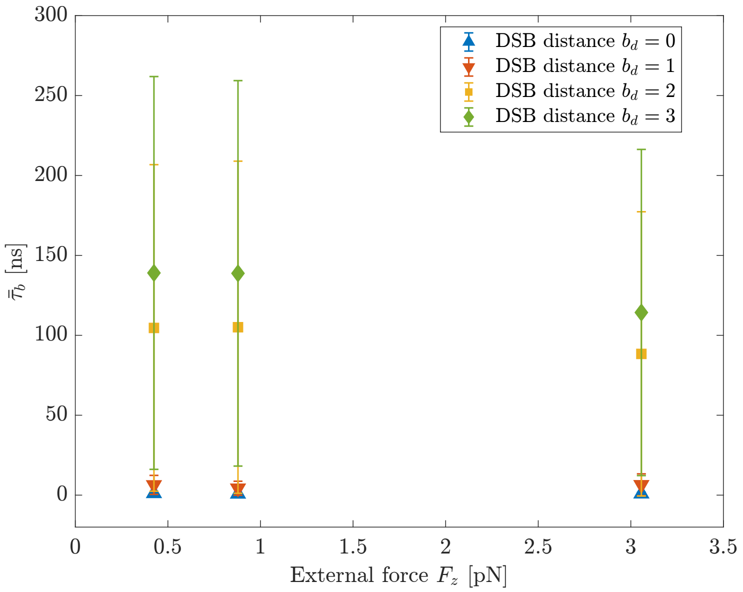

Supplementary Figure S10: Zero-force extrapolation of the characteristic rupture times of DNA by the diverse DSB motifs, reporting of a negligible effect of F_z in the low-force regime.

Supporting References

[1] Carlos J Bustamante et al. "Optical tweezers in single-molecule biophysics". In: *Nature Reviews Methods Primers* 1.1 (2021), p. 25.

[2] Simona Cocco, Rémi Monasson, and John F Marko. "Force and kinetic barriers to unzipping of the DNA double helix". In: *Proceedings of the National Academy of Sciences* 98.15 (2001), pp. 8608–8613.

[3] Olga K Dudko, Gerhard Hummer, and Attila Szabo. "Intrinsic rates and activation free energies from single-molecule pulling experiments". In: *Physical review letters* 96.10 (2006), p. 108101.

[4] E. Evans. "Probing the relation between force—lifetime—and chemistry in single molecular bonds". In: *Annual review of biophysics and biomolecular structure* 30 (2001), pp. 105–128.

[5] John F. Marko and Eric D. Siggia. "Stretching DNA". In: *Macromolecules* 28.26 (1995), pp. 8759–8770.

[6] Michelle D Wang et al. "Stretching DNA with optical tweezers". In: *Biophysical journal* 72.3 (1997), pp. 1335–1346.
